# Review of Thin Lithium Metal Battery Anode Fabrication – Microstructure – Electrochemistry Relations

**DOI:** 10.1002/adma.202511817

**Published:** 2025-10-13

**Authors:** Yuhang Hu, Zidong Chen, Yixian Wang, Haorui Hou, Bingcheng Chen, David Mitlin, Wei Liu

**Affiliations:** ^1^ Institute of New‐Energy and Low‐Carbon Technology (INELT) College of Carbon Neutrality Future Technology Sichuan University Chengdu Sichuan 610065 China; ^2^ State Key Laboratory of Intelligent Construction and Healthy Operation and Maintenance of Deep Underground Engineering Sichuan University Chengdu Sichuan 610065 China; ^3^ Materials Science and Engineering Program & Texas Materials Institute The University of Texas at Austin Austin TX 78712 USA

**Keywords:** electrochemical deposition, lithium alloys, lithium metal anode, lithium metal battery, thin foil

## Abstract

While lithium metal foils used for research may be upward of 250 µm in thickness, anodes for viable lithium metal batteries (LMBs) must be at least one order of magnitude thinner. This review focuses on fabrication approaches that promise to bridge this divide, highlighting the known/unknown processing – microstructure – electrochemical properties interrelations. Four general methodologies are discussed, starting with metallurgical ingot extrusion and rolling, followed by solidification casting, solution‐based wet methods, and physical vapor deposition (PVD). Each section begins with an outline of the underlying principles of the approach and how this limits the minimal thickness, morphology, bulk microstructure, and surface chemistry of the resultant anodes. The discussion then moves to specific case studies that illustrate how various state‐of‐the‐art research efforts have overcome these limitations by employing a range of strategies that include alloy and composite metallurgies, functionalized current collector coatings, and liquid‐phase additives. It is highlighted that methodologies resulting in planar and conformal lithium films, and subsequently improving electrochemical performance, are fairly consistent across all four fabrication classes. Each section concludes with a critical discussion of the research necessary to advance the field, identifying key outstanding scientific questions and “unknowns.”

## Graphical abstract

Thin, lightweight lithium‐metal anodes are pivotal for practical high‐energy batteries. This review surveys processing routes that convert diverse Li precursors, e.g., ingots, melts, solutions, and vapor, into Li‐rich foils with controlled thickness, areal density, and tailored functionality. The fabrication‐microstructure‐electrochemistry linkage is clarified, and distill design rules for optimizing performance in both liquid‐ and solid‐state Li‐metal batteries.

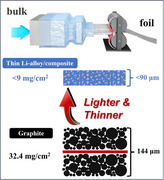

## 1 Introduction

**Figure 1:**
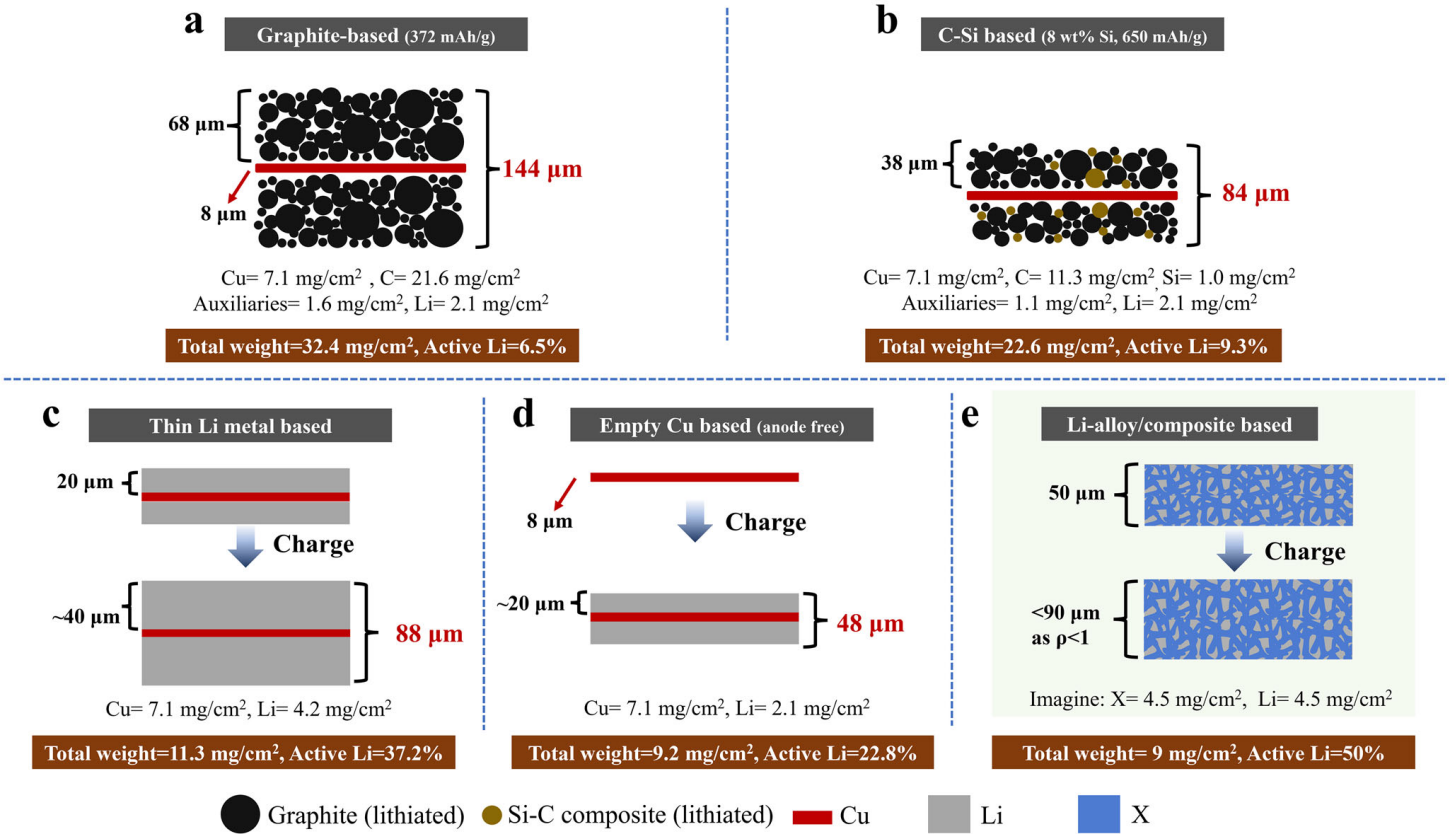
The comparison of various anodes with pragmatic cell area capacity (4 mAh cm^−2^). a) Graphite; b) C‐Si composite; c) thin Li metal; d) “anode‐free”; e) self‐standing Li‐alloy/composite foil. Note the total weight indicates the extent of the anode's lightweight, and the active Li percentages represent the efficiency in storing Li (and hence the energy); both aspects are reified embodiments of cell energy density and cost.

Graphite as an anode material dominates in the battery industry due to its superior safety and cycle life; however, it only offers a limited Li‐storage capacity of 372 mAh g^−1^ due to LiC_6_ stoichiometry. The consensus is that processing graphite into spherical particles with a diameter preferably in the range of 5–20 µm is optimum, with larger particles being solid‐state diffusion‐limited during lithiation.^[^ ^1^, ^2^ ^]^ **Figure** **1** provides a comparison of various anodes with pragmatic cell area capacity (4 mAh cm^−2^). Going from (a–d), the commercial readiness of these cells decreases, while the achievable capacity increases. Figure 1a shows a conventional commercial graphite anode, while 1b shows a Si─C composite that has also been commercialized at large scales. Figure 1c,d presents anodes based on thin Li metal foil, i.e., LMA., and on an anode‐free (AF) configuration employing an “empty” Cu current collector opposing a lithiated cathode. Pairing a graphite anode with a ceramic cathode such as LiNi_x_Co_y_Mn_1‐x‐y_O_2_ (NCM) or LiFePO_4_ (LFP) represents the mainstream LIB configuration. Such cells display a specific energy in the range of 150–250 Wh kg^−1^, depending on the cathode employed. Unlike carbons with the 372 mAh g^−1^ Li‐storage capacity (LiC_6_), Si can store up to 3590 mAh g^−1^ of Li (Li_15_Si_4_), and it is naturally abundant in the earth's crust. However, single‐phase Si anodes have not reached commercial maturity due to several factors, including poor cyclability. Rather, composites of Si and carbon have achieved large‐scale commercial maturity.^[^ ^3^, ^4^ ^]^ Figure 1b illustrates Si─C composite anodes with a specific capacity of 650 mAh g^−1^ (Si: C = 8: 92 wt.%), showing reduced electrode weight by ≈30% (from 32.4 to 22.6 mg cm^−2^) and electrode thickness by ≈42% (from 144 to 84 µm) as compared to the graphite counterpart. Paired with a high‐Ni NCM cathode, such a Si─C composite can enable cell energy densities up to 300–350 Wh kg^−1^.^[^ ^5^ ^]^

An alternative anode configuration relies on coating 20 µm‐thin Li metal onto Cu foil, i.e., the Li‐Cu composite foil. This is illustrated in Figure 1c. Such electrode configuration results in the reduction of electrode weight by ≈65% (from 32.4 to 11.3 mg cm^−2^) and thickness by ≈39% (from 144 to 88 µm) as compared to graphite. Numerous recent LMB advances reporting pouch‐cells cycle life rely on such anode technology, where specific energy of >400 Wh kg^−1^ and extended cycle life of > 300 are achieved at full charge–discharge cycles.^[^ ^6^, ^7^, ^8^ ^]^ Figure 1d illustrates an anode‐free lithium metal battery (AF‐LMB) cell configuration that employs an “empty” Cu current collector to oppose a cathode that is the only source of Li ions. This electrode design results in the reduction of electrode weight by ≈71% (from 32.4 to 9.2 mg cm^−2^) and thickness by ≈67% (from 144 to 48 µm) as compared to graphite. Anode‐free cell configuration with NCM811 cathode has been demonstrated to achieve an energy of upward of 500 Wh kg^−1^. However, this design is extremely challenging since employing conventional Cu current collectors and electrolytes results in rapid capacity loss and metal dendrite growth.^[^ ^9^, ^10^, ^11^, ^12^ ^]^ Readers are encouraged to consult prior review articles that focus on AF‐LMBs.^[^ ^13^, ^14^, ^15^ ^]^ While going AF gives the potential to have a transformative energy, cost, and (possibly) safety improvement, to our knowledge, there are no existing commercial embodiments of an AF‐LMB employing a liquid electrolyte.

Replacing traditional graphite anodes with metallic lithium metal anode (LMA) represents an avenue to achieve greater than 500 Wh kg^−1^ specific energy on a cell level since the capacity of pure lithium is ten times higher than that of graphite (3860 mAh g^−1^ vs 374 mAh g^−1^).^[^ ^16^, ^17^, ^18^ ^]^ Moreover, employing an LMA enables the possibility of high‐energy and relatively inexpensive Li‐S batteries, where the S‐based cathode is not pre‐lithiated.^[^ ^19^, ^20^, ^21^, ^22^, ^23^ ^]^ Several approaches have been explored to tune the electrodeposition/dissolution of Li to enhance electrochemical cycling and suppress dendrites. Notable examples include the fabrication of lithiophilic current collectors,^[^ ^24^, ^25^ ^]^ more Li‐compatible electrolytes,^[^ ^26^, ^27^ ^]^ artificial protective membranes,^[^ ^28^, ^29^ ^]^ and advanced charge‐discharge protocols.^[^ ^30^ ^]^ It is worth noticing that many existing studies adopt laboratory Li discs ≈500 µm in thickness for cell evaluation.^[^ ^31^, ^32^, ^33^, ^34^ ^]^ This is equivalent to 100 mAh cm^−2^ of excess capacity, which can mask issues such as non‐ideal cycling Coulombic efficiency (CE).^[^ ^35^, ^36^, ^37^ ^]^ An excessively thick Li metal anode subtracts from the gravimetric and volumetric energy of the cell, as well as increases the cost and possibly reduces the safety since the metal can burn. Closing the gap between seemingly well‐performing lab‐scale cells with excessive Li and practical implementation‐ready LMBs represents a major challenge.^[^ ^38^ ^]^ Commercial high‐energy LIBs adopt areal loadings of ≈4 mAh cm^−2^ on the cathode side, pairing with a graphite anode with ≈84 µm in thickness (double side). Since active Li ions are stored in the discharged cathode, in principle, the Li present at the anode is only there to make up for the ongoing Coulombic efficiency (CE) losses during cycling. There is no need to balance capacities, e.g., employing even a 20 µm thick foil to counter the ≈4 mAh cm^−2^ cathode. However, as will be discussed, exceedingly thin foils are difficult to achieve by conventional metallurgical methods. Industrial consensus, therefore, focuses on 20 µm as an appropriate development target, with thinner foils being generally considered as desirable. Quotative analysis of how the Li foil thickness affects the cell energy may be obtained from the Argonne National Laboratory BatPaC: Battery Manufacturing Cost Estimation model.^[^ ^39^ ^]^

The inherently chemically reactive, mechanically soft, and tribologically sticky properties of metallic Li pose major challenges to thin LMA fabrication. Conventional extrusion rolling‐based techniques produce ≈150 µm Li foils. Further thinning of Li foil relies on the introduction of lubrication oil, or a mechanical substrate that prevents the roller‐sticking.^[^ ^40^ ^]^ This leads to an inevitable compromise of surface purity or foil weight if a Cu backing is used. It is also known that the cost of thin (< 50 µm) Li foil fabrication by such proven techniques is upward of 1 000 USD kg^−1^, significantly more expensive than for the starting Li‐metal ingots that are less than 200 USD kg^−1^ (as of December 2024).^[^ ^40^ ^]^ Lithium foil properties appear to be strongly manufacturer‐dependent, being correlated with manufacturing methods such as pretreatment, as well as handling and storage conditions.^[^ ^41^ ^]^ Variations in surface chemistry as well as in the bulk‐phase microstructure (grain size/distribution, porosity, texture, surface roughness, inevitable alloying) of as‐fabricated foils are key drivers for the wide variation reported in anode performance. This will be a key theme for discussion throughout the manuscript.

A great deal of effort is being devoted to tackling the difficulties in processing thin LMAs. One of the effective approaches is modifying metallic Li with alloying elements such as Sn, In, Zn, Mg, and Ca,^[^ ^42^, ^43^, ^44^, ^45^, ^46^ ^]^ intending to achieve improved processability. The approach may involve tuning the microstructure of body‐centered cubic (*bcc*) Li to form solid solutions and/or to nucleate second‐phase intermetallics.^[^ ^36^, ^47^, ^48^ ^]^ A solid solution and/or densely distributed second‐phase intermetallic will improve the mechanical properties of the Li foils, nominally through accelerated work‐hardening, which in turn promotes greater foil formability and resistance to necking. Such alloying strategies open up wide opportunities for tuning the electrochemical behavior of the resultant anode.^[^ ^49^, ^50^, ^51^, ^52^, ^53^, ^54^, ^55^, ^56^, ^57^ ^]^ This will be discussed in detail throughout the manuscript. Beyond the conventional extrusion‐rolling methods, there is the emergence of alternative LMA fabricating methods, including solution–precipitation and vapor deposition.^[^ ^58^, ^59^ ^]^

This review aims to provide a critical analysis of the established and emerging methodologies for thin LMAs. Prior reviews on Li metal have focused on the varying aspects of LMA and LMBs, including the lab‐scale preparation and scale‐up of LMAs,^[^ ^60^ ^]^ advanced liquid electrolytes,^[^ ^61^, ^62^ ^]^ or artificial membrane separators,^[^ ^63^ ^]^ and modified current collectors,^[^ ^64^, ^65^ ^]^ all of which give rise to improved electrochemical LMBs performance. In each case, crucial features of the design that are responsible for inhibiting Li dendrites and promoting a stable solid electrolyte interphase (SEI) were identified. Building upon existing knowledge, this review aims to provide a critical analysis of the established and emerging methodologies for fabricating thin LMAs. It appears that electrochemical stabilization of thin (< 50 µm) LMAs is substantially more challenging than that of conventional (≈500 µm) laboratory‐thick Li discs.^[^ ^66^, ^67^ ^]^ As will be discussed, the processing necessary to achieve “thin” Li metal results in major modifications in both the bulk and the surface microstructure of the foils.

Over the past decade, composite Li/Li‐alloy anodes have garnered extensive attention. As illustrated in Figure 1e, one may reasonably project that a Li‐rich alloy or composite foil that possesses >50% Li concentration at fully charged state, enables effective >70% weight reduction in the weight of the anode as compared to the weight of a typical graphite electrode with 4 mAh cm^−2^ areal capacity (9 mg cm^−2^ vs ≈ 32 mg cm^−2^). This anode configuration can be a pivotal component for the development of >500 Wh kg^−1^ cells. Progressive thinning of Li metal anodes, including with utilization of alloy or composite foils, is a key focus for achieving high‐energy metal batteries that are safe and economical. Hence, the design, fabrication, and implementation of thin LMAs emerge as a pressing need. Several scientific and engineering questions require further exploration, such as what is the ideal processing route to achieve sub‐20 µm foils? How do such thin LMAs differ from conventional 500 µm laboratory Li discs in microstructure and electrochemical functionality, and why? In the following sections, we will elaborate on the recent advances in thin LMA fabrication. Case‐by‐case analysis will elucidate the “known” processing‐microstructure‐property relations while identifying the key “unknowns” that require further analysis by the community.

This review is structured in sections that each focus on one major fabrication route, the resultant microstructure of the LMA, and the associated electrochemical performance. We categorize the fabrication routes into four broad categories based on the physical process: ingot extrusion and rolling, solidification casting, solution‐based wet methods, and physical vapor deposition (PVD). For each case, we appraise the merits and the intrinsic limitations of each method. In parallel, microstructural features and electrochemical metrics for each approach are presented and linked to the synthesis approach. Throughout the review, we provide a forward‐looking perspective that distills the existing state‐of‐knowledge into a strategic roadmap for future research.

## 2 Ingot Extrusion and Rolling

### 2.1 Creep Deformation of Lithium Metal

Creep deformation becomes important above a homologous temperature (operating temperature divided by melting temperature, both in Kelvin) of 0.4. If creep deformation is active, the material will display time‐dependent plastic deformation at all applied stress levels, including substantially below the nominal yield stress. The material will plastically deform with any applied force if one just waits long enough. Metals typically exhibit diffusional creep at low stress, and dislocation climb‐controlled (pure metals, alloys) or dislocation glide (alloys only), controlled power law creep at intermediate and high stresses. The relevant stress exponent is 1 for Coble and Nabarro–Herring creep, ≈3 for dislocation glide power law creep, and ≈5–7 for dislocation climb power law creep.^[^ ^74^, ^75^, ^76^ ^]^ Creep deformation can be controlled, however, with solid solution elements and precipitate phases, where climbing and gliding dislocations become pinned by these heterogeneities.^[^ ^77^ ^]^ Solid solution and precipitation strengthening will then promote work hardening by pinning gliding and climbing dislocations. This significantly aids forming operations, allowing greater plasticity without necking and thinner achievable dimensions. The traditional industrial method for producing Li metal foils is some variation of mechanical extrusion followed by rolling deformation at near ambient temperature. The achievable lithium thickness depends on parameters such as extrusion pressure, speed, and Li billet temperature, as well as lubrication conditions. The final thinning process relies on mechanical rolling. When the targeted thickness is below 100 µm, uniform rolling becomes technically challenging due to the foil's poor work hardening (leading to necking) and its propensity to mechanically stick to roller surfaces. Lithium metal is known for its low yield strength (≈2 MPa) and low hardness (≈5 MPa).^[^ ^70^, ^78^ ^]^ Moreover, at 25 °C, the homologous temperature of Li metal is 0.67, resulting in extensive diffusional creep even below the yield stress.

**Figure 2:**
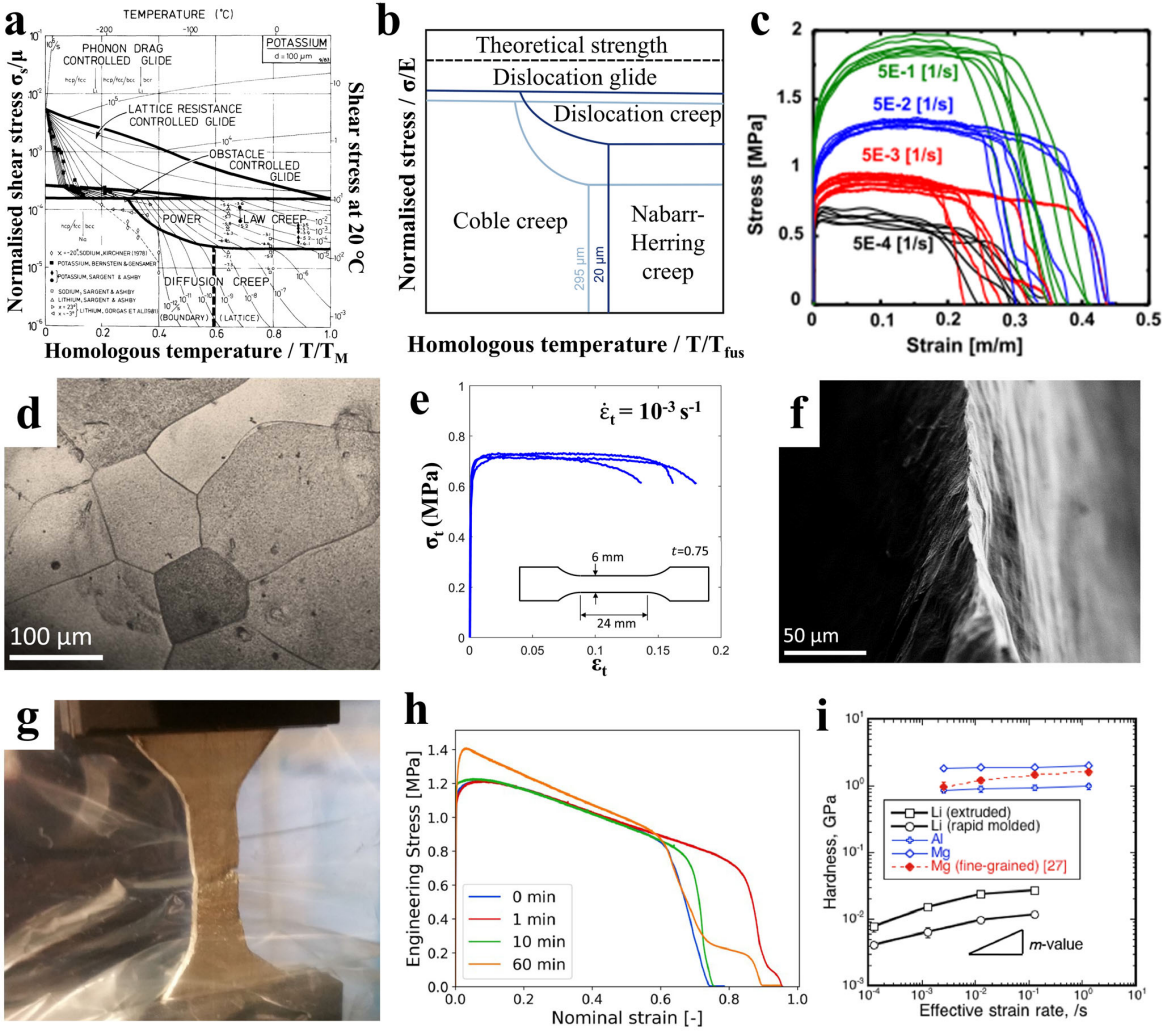
a) Classical Ashby deformation mechanisms map for alkaline metals as a function of homologous temperature, i.e., T/T_melting_ in Kelvin,^[^ ^68^ ^]^ b) Recently published deformation map specifically for Li metal, indicating that at near room temperature plasticity occurs by diffusional creep at low stresses and by dislocation climb creep at higher stresses.^[^ ^69^ ^]^ c) Room temperature stress‐strain data for Li metal in tension, displaying strain rate/time dependence of plastic behavior, directly indicative of creep.^[^ ^70^ ^]^ d) Light optical microscope image of the grain structure of an as‐cast lithium sample. e) The true stress‐true strain response of cast Li metal. f) SEM image of the 2D necked region of the failed specimen.^[^ ^71^ ^]^ g) Fracture initiation at an uniaxial tension specimen during a test in an argon‐filled Ziploc plastic bag; h) Uniaxial tension tests at different times of air exposure before tensile testing, indicating the role of surface oxidation in Li metal ductility.^[^ ^72^ ^]^ i) The variation of indentation hardness as a function of effective strain rates in the Li vs pure Al and Mg. Panel a reproduced with permission: Copyright 1984, Pergamon Press. Panel b reproduced with permission: Copyright 2023, Wiley. Panel c reproduced with permission: Copyright 2020, Elsevier. Panels d to f reproduced with permission: Copyright 2023, Elsevier. Panels g and h reproduced with permission: Copyright 2021, Elsevier. Panel i reproduced with permission: Copyright 2023, Elsevier.^[^ ^73^ ^]^

**Figure** **2**a shows a classical (published in 1984) Ashby deformation mechanisms map for alkaline metals as a function of homologous temperature, i.e., T/T_melting_ in Kelvin.^[^ ^79^ ^]^ Figure 2b displays a recently published deformation map specifically for Li metal, indicating that at near room temperature, plasticity occurs by diffusional creep at low stresses and by dislocation climb creep at higher stresses.^[^ ^69^ ^]^ From both figures, it is evident that creep is the dominant plasticity mechanism at room temperature. From the maps, it is evident that a thermal (non‐thermally activated) dislocation glide is not a relevant deformation mechanism. This indicates that conventional work hardening, present due to dislocations piling up against grain boundaries, twins, or other dislocations, should be minimal for Li deformed at or above room temperature. Moreover, according to the standard understanding of creep deformation, a smaller grain size will accelerate the creep rate by providing a fast path for metal atom diffusion, and by acting as a source of vacancies.

Figure 2c shows room temperature stress–strain data for Li metal in tension, displaying strain rate/time dependence of plastic behavior, which is directly indicative of creep.^[^ ^70^ ^]^ For a more in‐depth analysis of creep deformation in Li, the reader is guided toward an excellent recent review by Kim et al.^[^ ^80^ ^]^ Figure 2d displays a light optical microscope image of the grain structure of an as‐cast lithium sample, and the diameter of the single grain surpasses 100 µm. Figure 2e shows the true stress‐true strain response of cast Li metal. Figure 2f displays a cross‐section SEM image of the 2D necked region of the failed specimen, which shows a narrow and sharp fracture surface.^[^ ^71^ ^]^ Figure 2g displays the fracture initiation at a uniaxial tension specimen during a test in an argon‐filled Ziploc plastic bag. Figure 2h displays uniaxial tension tests at different times of air exposure before tensile testing, indicating the role of surface oxidation in metal ductility.^[^ ^72^ ^]^ This indicates that even limited exposure of Li surfaces to air will influence the foil ductility during rolling operations, potentially creating quality control problems, especially when aiming for thinner foils with higher surface‐to‐volume ratios. With pure Li foils, room‐temperature creep can lead to premature necking, since work hardening is inhibited due to a lack of dislocation pinning sites. In addition to dynamic annealing through creep, pure Li foils are likely to dynamically recrystallize during rolling operations and undergo dynamic grain growth.^[^ ^81^, ^82^ ^]^ Both would further inhibit work hardening, which is necessary to prevent necking during rolling operations. Figure 2i shows the variation of indentation hardness as a function of effective strain rates in the Li vs pure Al and Mg. These results demonstrate that regardless of the processing method (extrusion vs. rapid molding) and the associated microstructure, Li is both extremely soft and sensitive to strain rate.^[^ ^73^ ^]^ In summary, rolling of pure Li into progressively thinner formats is challenging due to poor work hardening as a result of extensive creep, creating the propensity for necking and sheet rupture/tearing.

The softness of pure Li foils will also lead to their mechanical sticking to the asperities in the rollers, unless the roller surfaces are impregnated with lubricating oil or a polymer coating. It should be pointed out that the roller sticking issues widely reported in the literature are likely mechanical in origin, rather than due to a reactivity between the roller and the foil. The roller metallurgy is nearly always some form of stainless steel, which at ambient conditions is covered by a ≈10 nm (total) bilayer of chromium oxide. The outer oxide is more oxygen‐rich, being Cr_2_O_3_ with a corundum structure, while the inner oxide is Cr_3_O_4_ with a spinel structure. It is known that the standard enthalpy of formation for Cr_2_O_3_ is ≈−1140 kJ m^−1^, while for Cr_3_O_4_ it is −1134 kJ mol^−1^. The standard enthalpy of formation of lithium oxide Li_2_O is ≈−596 kJ mol^−1^. The standard enthalpy of formation for lithium carbonate Li_2_CO_3_ is −1216 kJ mol^−1^. Therefore, it is expected that the oxide/carbonate‐terminating Li surface does not react with the chromium oxide‐terminating stainless‐steel surface. However, as the rollers’ surfaces cannot be machined to be perfectly smooth, during rolling operations, the soft Li metal is physically smeared into the grooves, causing mechanical adhesion. Because of the superior thermodynamic stability of Cr_2_O_3_/Cr_3_O_4_, Li metal can not reduce chromium oxide. If rollers made from plain steel were to be employed instead, the terminating surfaces would be covered by a layer of rust. The standard enthalpy of formation for Fe_2_O_3_ and Fe_3_O_4_ is −824 and −1118 kJ mol^−1^, respectively. Therefore, a reaction between Li foils and conventional steel is also unlikely. The oil‐based and polymer coating employed to allow for improved rolling of Li foils serves to reduce friction and smooth the roller surfaces. Importantly, these incompressible lubricants fill the asperities, preventing Li penetration.

### 2.2 Mechanical Rolling of Lithium Foils

As an example of state‐of‐the‐art industrial Li rolling mills, e.g., as offered by REDEX group (https://www.redex‐group.com/prm/plf/), achieve typical foil thicknesses of 200–500 µm with a thickness control to 1 µm and roll bending to eliminate edge cracking. There are also scientific reports of Li foils rolled down to 20 µm thickness, obtained by the introduction of lubricant oil on the roller surface, or by involving a plastic release layer with lubricative surface coatings.^[^ ^40^, ^86^ ^]^ However, this strategy can contaminate the metal surface with residual lubricant/polymer, thereby affecting the foil's electrochemical performance. For example, recent studies have shown that properly cleaning the post‐rolled foil surface leads to more favorable electrochemical properties.^[^ ^39^ ^]^ Other notable examples of innovative rolling methodologies are presented in Figure 1 and discussed below.

**Figure 3:**
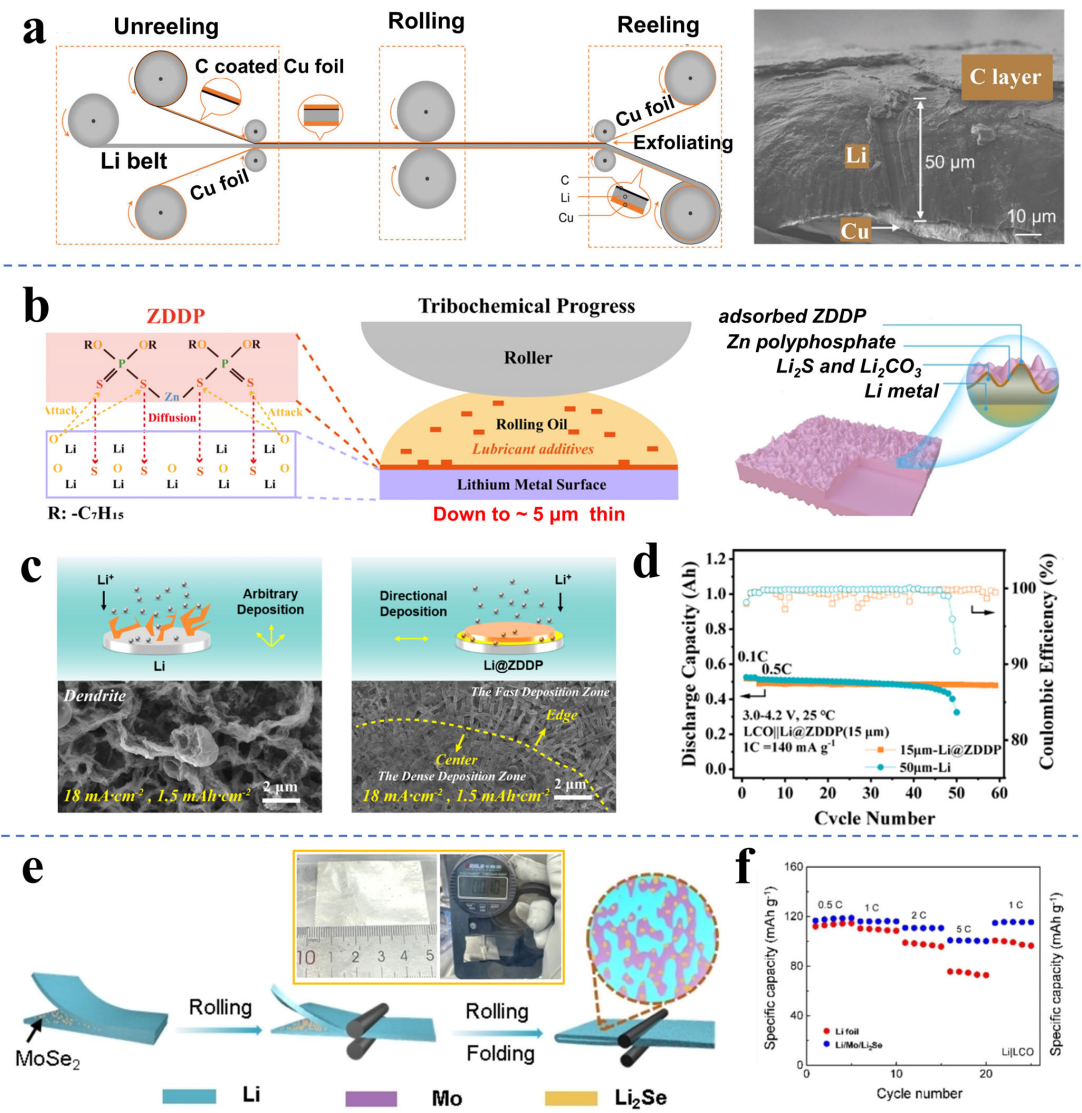
a) Carbon‐coated Cu foil enables roll‐to‐roll fabrication of 50 µm Li directly connected to the opposing Cu foil, with the carbon layer bonded to its opposite surface.^[^ ^83^ ^]^ b) Schematic illustration of lubricant oil containing zinc dialkyl dithiophosphate (ZDDP) forming a rigid surface layer on the Li surface, enabling foil rolling down to 5 µm. c) Schematics and associated SEM images of Li electrodeposits formed on the ZDDP‐modified Li and the baseline Li, respectively. d) Electrochemical performance of LCO‐based pouch cells, employing ZDDP‐modified Li and the baseline Li.^[^ ^84^ ^]^ e) Schematic of a MoSe_2_‐derived interface enabling the large‐area rolling of modified Li‐foil down to 10–40 µm, the MoSe_2_ reacts with Li to give an interlayer comprising Mo and Li_2_Se. f) Rate performances of Li||LCO cells employing MoSe_2_ modified Li and neat Li as anodes. Panel a reproduced with permission: Copyright 2019, Springer. Panels b to d reproduced with permission: Copyright 2023, Springer Nature (open access). Panels e and f reproduced with permission: Copyright 2024, Wiley.^[^ ^85^ ^]^

Zhang et. al. developed a “skin‐grafting” strategy that enables roll‐to‐roll manufacturing of ≈50 µm Li foils.^[^ ^83^ ^]^ This approach is illustrated in **Figure** **3**a. A thin carbon (≈3 µm) coating of the Li metal surface prevented the Li metal from sticking to the rollers and ultimately improved the foil's electrochemical performance. The as‐fabricated sandwiched Li/C composite anodes exhibited improved cycling stability both in Li | LiFePO_4_ and Li | S coin cells (over 100 cycles) and in pouch cells. A discussion of how carbon supports and interlayers affect Li metal electrodeposition/dissolution is also provided in reference.^[^ ^9^ ^]^ It should be noted that the large‐scale fabrication of carbon‐based films coupled with Cu foils remains a major engineering challenge. The now rarely utilized processes for roll‐to‐roll manufacturing of photographic films may be one potential source of knowledge and technology transfer, as the commercial process is based on thin film coating of carbon‐containing slurries onto flexible, large‐area supports.

Chen's group reported that zinc dialkyl dithiophosphate (ZDDP), an anti‐wear lubricant, can facilitate the rolling of ultrathin Li foil.^[^ ^84^ ^]^ Those results are illustrated in Figure 3b,c. An in situ reaction was identified to occur during the rolling process, which generated an interfacial protective layer, comprising an organic polyphosphate and inorganic Li_2_S and Li_2_CO_3_. It was argued that the hybrid organic‐inorganic layer functioned as an artificial SEI layer that passivated the Li surface and enhanced electrochemical stability. The fabricated Li@ZDDP foil was able to achieve stable Li deposition even at an extreme current density of 18 mA cm^−2^, 1.5 mAh cm^−2^ in half‐cells, as shown in Figure 3c. Per Figure 3d, a pouch cell employing a 15 µm thick Li@ZDDP anode (N/P = ≈1.2) exhibited stable cycling over 60 cycles, while the control group employing a commercial 50‐µm‐thick Li anode declined after ≈45 cycles. In another study by the same group, a fluoro‐polymer grease was shown to serve as a fine lubricant for the thinning of Li. The grease reacted with the Li surface to form a 2 µm thick interlayer, consisting of a LiF/LiC_6_ inorganic framework hybridized with ‐CF_2_‐O‐CF_2_‐ organic species.^[^ ^87^ ^]^ This layer greatly improved the Li deposition uniformity, enabling full Li‐S pouch cells under a lean electrolyte of 3.3 µL mg^−1^.

Xia et al. developed a 10–40 µm thick Li/Mo/Li_2_Se composite foil by cold‐rolling MoSe_2_ powder into Li metal.^[^ ^85^ ^]^ As illustrated in Figure 3e, this enabled the fabrication of large‐area foils with enhanced malleability and mechanical strength. It was argued that the in situ generated Li_2_Se and Mo nanoparticles enhance Li‐ion transport through the interface. As illustrated in Figure 3f, this enabled the reversible cycling of a full cell with an LCO cathode at rates up to 5C. The NCM811‐based pouch cells deliver an energy density of 329.2 Wh kg^−1^, and perform stable 25 cycles under a low N/P ratio of ≈1.2 and lean electrolyte of ≈0.95 g Ah^−1^. While the Li‐based control pouch cells degraded after 5 cycles under the identical conditions.

### 2.3 Precipitation and Composite Strengthened Lithium

Lithium metal has been alloyed with Sn,^[^ ^88^ ^]^ Sb,^[^ ^89^ ^]^ In,^[^ ^90^ ^]^ Al,^[^ ^91^ ^]^ Te,^[^ ^92^ ^]^ Hg,^[^ ^48^ ^]^ which resulted in the formation of dislocation pinning intermetallic precipitates. The alloying approach has led to improved processability and enhanced electrochemical behavior (these effects are not directly correlated). Studies also report directly incorporating compounds such as LiF^[^ ^93^, ^94^, ^95^ ^]^ or Li_2_Se^[^ ^96^, ^97^ ^]^ and introducing metal fluoride or selenides^[^ ^85^, ^98^ ^]^ to enhance Li foil fabrication. When present in sufficient volume fraction, the intermetallic phase reinforcements will also lead to higher elastic stiffness of the foils. Elements forming random or ordered solid – solutions, such as Ag,^[^ ^52^ ^]^ are also effective in pinning dislocations and promoting creep strength. It should be pointed out that the elastic stiffness of alloyed or precipitation‐strengthened foils will be similar to that of pure Li (Young's modulus is minimally affected); only higher volume fraction multi‐phase composites could be stiffer. Moreover, it is not obvious that a higher elastic stiffness of the foil is actually beneficial for either processability or electrochemical performance.

**Figure 4:**
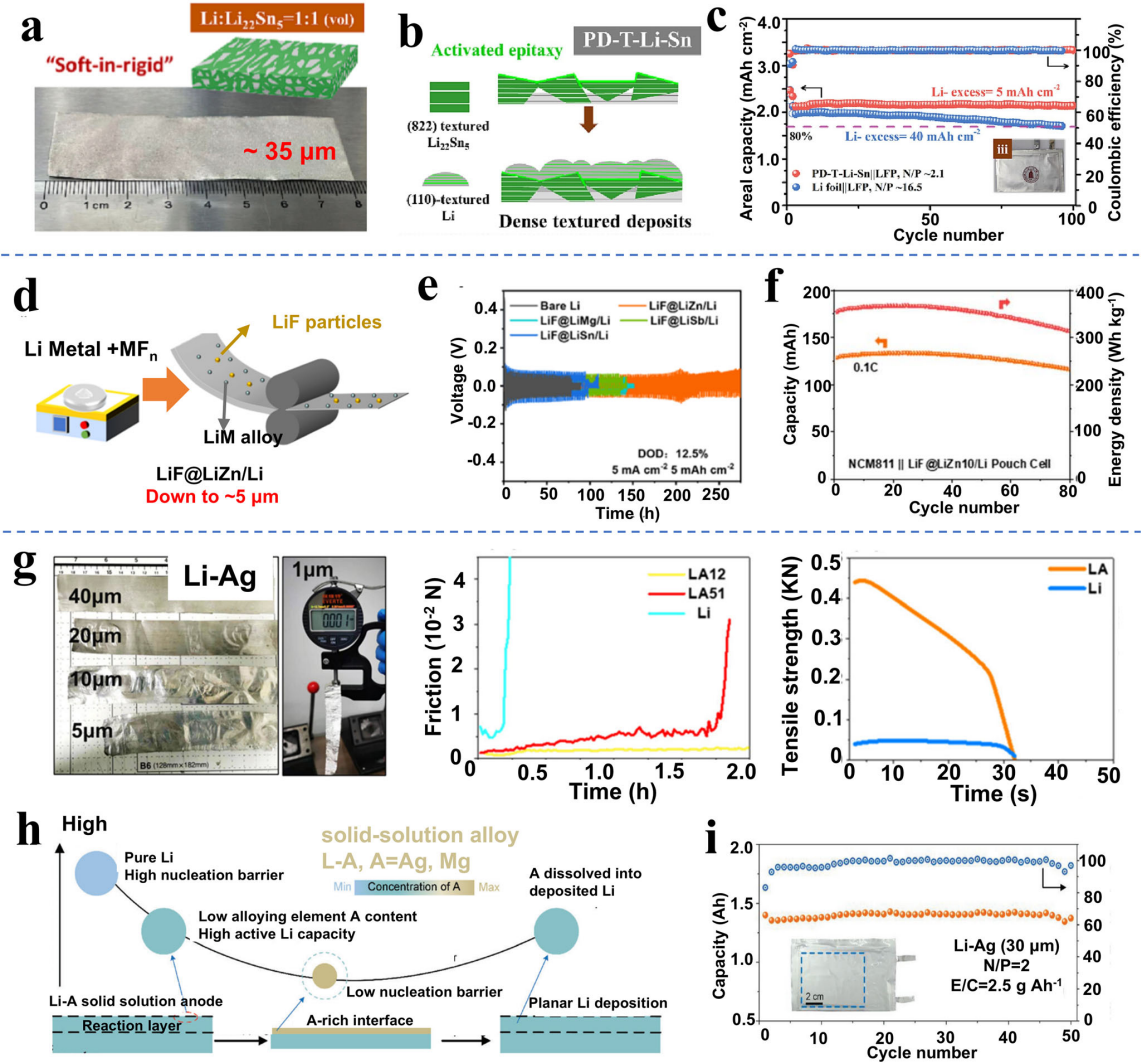
a) Schematics of obtaining a malleable alloy for foil rolling by doping Li with 8.9 at% Sn, b) The rolling‐induced (822)‐texture in Li_22_Sn_5_, conducive to (110)‐preferred Li‐electrodeposition, c) Performances of pouch cells employing 200 µm Li and 50 µm thin Li‐Sn foils, where the later shows improved cycle stability under lower NP ratios and less Li‐excess.^[^ ^88^ ^]^ d) Schematics of incorporating metal fluorides into Li metal to yield modified Li‐alloy foils, e,f) Operation of symmetric cells and full pouch cells employing 40 µm foils of metal fluorides modified Li, where ZnF_2_‐modification leads to superior plating‐stripping stability.^[^ ^99^ ^]^ g) Schematic of doping Ag into Li to yield a malleable alloy allowing cold rolling into thin foils with lower stickiness (middle) and higher strength (right).^[^ ^100^ ^]^ h) Schematic of Li‐Ag and Li‐Mg alloys allowing Li extraction and insertion via reversible solid solution reactions. i) Performances of Ah‐level pouch cells adopting 30 µm Li‐Ag (0.7 at% Ag) foil anodes. Panels a, b and c reproduced with permission: Copyright 2023, Elsevier. Panels d, e and f reproduced with permission: Copyright 2024, The Royal Society of Chemistry. Panel g reproduced with permission: Copyright 2022, The Royal Society of Chemistry. Panels h and I reproduced with permission: Copyright 2023, Wiley.^[^ ^101^ ^]^

Liu et al. co‐rolled Li metal and ≈9 at.% Sn to create an equal volume Li‐Li_22_Sn_5_ dual‐phase alloy, which was in accordance with the equilibrium binary phase diagram.^[^ ^88^ ^]^ Per **Figure** **4**a, the dual‐phase alloy displayed enhanced malleability, allowing thin foil fabrication down to 35 µm. During rolling, the mechanically hard Li_22_Sn_5_ intermetallic particles promoted work hardening (creep strength) in the Li metal. During subsequent electrochemical testing, the Li_22_Sn_5_ “skeleton” enhanced the electrodeposition and dissolution uniformity of the Li metal, promoting enhanced stability. The authors identified a strong rolling‐incurred (822) preferred orientation of the Li_22_Sn_5_ relative to the foil surface. This correlated with enhanced (110) preferred orientation on the Li electrodeposits, which in turn is known to correlate with improved electrochemical stability.^[^ ^9^ ^]^ The fabricated foils enabled LFP‐based pouch cells (N/P = ≈2.1) with 99% capacity retention after 100 cycles while employing conventional 1 M LiPF_6_ in carbonate electrolytes, per Figure 4b,c.

Wang et al. introduced 10 wt.% ZnF_2_ into Li metal and prepared 5–50 µm LiF@LiZn/Li foils.^[^ ^99^ ^]^ Those results are shown in Figure 4d. The LiF@LiZn/Li foil displayed greatly improved mechanical properties as well as enhanced stability in air. The role of various second phases, such as MF_n_, was discussed concerning the symmetric cell electrochemical performance, per Figure 4e. Particularly, the LiF@LiZn/Li foil with ZnF_2_ demonstrated the longest cycle life of 270 h as symmetric cells (cycling under 5 mA cm^−2^, 5 mAh cm^−2^), whereas cells with bare Li only lasted for less than 100 cycles. Utilizing 40 µm of such alloy foils, a pouch cell adopting NCM811 cathode (2.8 mAh cm^−2^, N/P = ≈2.5) showed >80% capacity retention after 80 cycles at 0.1 C full charge‐discharge. Those results are illustrated in Figure 4f.

As shown in Figure 4g, Zhang et al. prepared Li‐Ag solid‐solution foils with different thicknesses ranging from 40 to 1 µm.^[^ ^100^ ^]^ Silver alloying to the order of 1.3 at% (equal to 15.41 wt.%) resulted in a substantial improvement in the mechanical processability of the foils, as compared to unalloyed Li counterparts. Sun et al. explored the dynamic evolution and reaction mechanisms of Li‐Ag solid solution alloy foils, explaining the dendrite‐free, stable cycling performance. Those results are displayed in Figure 4h. It was demonstrated that the Ag‐Li solid solution reduced the nucleation barrier of electrodeposited Li and promoted enhanced wetting, therefore improving cycling performance. However, these electrochemical effects are distinct from the role of solid‐solution creep strengthening in enhancing the formability of the foils. Per Figure 4i, 1.4 Ah pouch cell with a 30‐µm‐thick Li‐Ag foil (N/P = 2) sustained 50 cycles with minimum capacity decay (< 5%).^[^ ^101^ ^]^

Li et al. reported the co‐doping of both Al and Mg elements into Li by melting‐rolling (the mass ratio of Li, Al, and Mg is controlled at 70:15:15).^[^ ^25^ ^]^ This approach combined intermetallic phase formation and solid solution strengthening. The resultant microstructure was likely a series of equilibrium (and possibly metastable) binary and ternary intermetallic phases, as well as a limited solid solution. The in situ formed Li─Mg solid solution and Li_9_Al_4_ skeleton possess improved lithiophilicity for Li plating, enabling the NCM811 full cell with a cathode loading mass of 20 mg cm^−2^ to exhibit excellent cycle stability for 100 cycles at 0.5C with a capacity retention of 90.8%. If the thickness of the alloy strip (≈100 µm) can be further reduced, the energy density of LMBs with Li─Mg─Al alloy will be more competitive. Chen et al. prepared a 5 µm Li─Mg─Al ternary alloy (96Li2Mg2Al) foil with excellent malleability in the rolling process.^[^ ^44^ ^]^ The ultrathin Li─Mg─Al foil exhibits a more interconnected ionic and electronic transporting network in the bulk phase.

All‐solid‐state batteries (ASSBs) using solid‐state electrolytes (SSEs) are another application where thin LMAs are required. A key bottleneck in the development of solid‐state batteries is the poor physical contact at the LMA and SSE interface, leading to current constriction. The non‐uniform electrodeposition/dissolution of LMAs that occur during cycling could generate interface gaps, especially at high current densities.^[^ ^102^ ^]^ Ding and co‐workers rolled a stack comprising sub‐50 µm lithium foil, LLTO solid electrolyte, and a poly(Ɛ‐caprolactone) interfacial layer to yield a 3D‐Li anode.^[^ ^103^ ^]^ Through a facile rolling‐press method, the inorganic/organic electrolyte frame could embed into the Li foil under rolling pressure due to the high ductility and elongation of Li‐metal. Coupled with a LiFePO_4_ cathode, ASSBs delivered stable cycling for 250 cycles at 1 C in coin cells. Earlier studies have shown some Li alloys exhibiting more favorable mechanical and/or electrochemical compatibility with SSEs vs pure Li foils. The alloys include In, Al, and Mg.^[^ ^102^, ^104^, ^105^, ^106^ ^]^ Zhang et al. prepared a 40‐µm‐thick Li─In alloy by compressing Li foil and In foil together at 760 MPa.^[^ ^107^ ^]^ An alloy foil with 2 wt.% In, that was paired with an NCM622 cathode (≈4 mAh cm^−2^) and Li_6_PS_5_Cl‐based electrolyte, was pressed a 760 MPa to form an integrated ASSB, showing a stable cycling capacity of up to 890 cycles.

Zhuang et al. argued that forming a porous structure in a Li‐alloy is beneficial for electrochemical stability, preparing and testing two types of Li‐Al alloy foils with varying porosity.^[^ ^108^ ^]^ The objective was to buffer electrodeposition/dissolution volume changes, thereby improving the anodes’ compatibility with the SSEs. Porous Li‐Al was prepared by compressing the mixture of Li powder and Al powder under 400 MPa, while dense Li─Al was obtained by pressing Li foil onto Al foil. Both alloys possessed a composition of Li to Al in a 1:1 molar ratio, which is equivalent to 79 wt.% Al in the foil. While porous Li─Al alloy showed a volume expansion of 17% upon lithiation (going from 72 to 84 µm), the dense Li‐Al of equal weight expanded 66% (from 56 to 93 µm). This is due to the porous structure suppressing the macroscopic volume changes that would occur during electrodeposition/dissolution of a dense foil. As with all such architectures, the volume changes are contained in the pre‐existing pores, albeit at the expense of having a lower overall electrode density. In a Li_6_PS_5_Cl‐based ASSB full cell (NCM811 cathode, 12.74 mg cm^−2^), the porous Li‐Al demonstrates 83% capacity retention after 1800 cycles. The full cells were cycled at 2.55 mA cm^−2^ and 15 MPa at room temperature. To provide an intuitive illustration of the electrochemical performances achieved by Li anodes fabricated by extrusion and rolling, we specifically compiled a concise table, as **Table** **1**.

**Table 1:** Summary of electrochemical performances of rolled thin Li metal anodes.

Materials type	Processing method	Electrochemical performances			Refs.
		Thickness (area capacity)	Half‐cell performances (electrolytes)	Full cell performance (cathode, N/P ratio, size)	
Li@ZDDP	Thinning Li metal with ZDDP lubricants	15–100 µm (3–20 mAh cm^−2^)	1.5 mA cm^−2^, 1.5 mAh cm^−2^, 2800 h, (1 M LiTFSI in DME/DOL 1:1 vol + 2% LiNO_3_)	Multi‐layer pouch cells, 100%@50 cycles, 0.5C; (≈2.7 mAh cm^−2^ LCO, **N/P =** 1.1, ≈5* × 8 cm^2^)	[84]
Li@CFO	Thinning Li metal with fluoropolymer grease	50–150 µm (10–30 mAh cm^−2^)	1 mA cm^−2^, 1 mAh cm^−2^, 5600 h (1 M LiTFSI in DME/DOL 1:1 vol + 2% LiNO_3_)	Coin cells 99.9%@450 cycles, 1C; (≈2.6 mAh cm^−2^ LFP, **N/P =** 3.8)	[87]
Li‐Sn	rolling Sn‐doped Li into self‐standing foils	35–100 µm (1.8–5 mAh cm^−2^)	1 mA cm^−2^, 2 mAh cm^−2^, 500 h (1 M LiPF_6_ in EC: DMC: EMC (1:1:1))	Single‐layer pouch cells ≈99.4%@100 cycles,0.5C (≈2.5 mAh cm^−2^ LFP, **N/P =** 2.1, ≈**4 ×4 cm^2^)**	[88]
LiAg	rolling Ag‐doped Li into self‐standing foils	1–40 µm (0.2–8 mAh cm^−2^)	1 mA cm^−2^, 1 mAh cm^−2^, 520 h (1 M LiPF_6_ in EC/DEC 1:1 vol + 10% FEC)	Multi‐layer pouch cells, 86%@100 cycles,0.1 C; (8 mAh cm^−2^ NCM811, **N/P =** 1.0, ≈**4 × 5 cm^2^**)	[100]
LiAg‐LiF	rolling Li metal and AgF particles into self‐standing foils	5–50 µm (0.9–9 mAh cm^−2^)	1 mA cm^−2^, 1 mAh cm^−2^, 500 h (1 M LiPF_6_ in EC/DEC (1:1) + 10% FEC+ 1% VC)	Single‐layer pouch cells ≈93.2%@35 cycles,0.25C; (3.4 mAh cm^−2^, LCO, **N/P =** 2.5, ≈**4.3 × 5.6 cm**)	[98]
LiZn‐LiF	rolling Li metal and ZnF particles	20–40 µm (3.5–7 mAh cm^−2^)	1 mA cm^−2^, 1 mAh cm^−2^, 1300 h (1 M LiTFSI in DME/DOL+2% LiNO_3_)	Single‐layer pouch cells 80% @ 80 cycles, 0.1 C (3.4 mAh cm^−2^, NCM811, **N/P =** 2.59)	[99]
Li/Mo/Li_2_Se	rolling Li metal and MoSe_2_ particle	10–40 µm (1.5–6 mAh cm^−2^)	3 mA cm^−2^, 1 mAh cm^−2^, 1500 h (1 M LiPF_6_ in EC/EMC (3:7))	Single‐layer pouch cells ≈90%@50 cycles,0.25C (5.3 mAh cm^−2^, LCO, **N/P =** 1.36)	[85]
Li‐Mg‐Ca	rolling Mg/Ca‐co‐doped Li metal	45 µm (5.9 mAh cm^−2^)	1 mA cm^−2^, 4 mAh cm^−2^, 1000 h (1 M LiTFSI in DME /DOL+1% LiNO_3_)	Multi‐layer pouch cells ≈80%@ 20 cycles (NCM811, 5.3 mAh cm^−2^, **N/P =** 1.8)	[109]
LiAl‐p	mixing and pressing equimolar Al and Li powder	≈70 µm (8 mAh cm^−2^)	0.5 mA cm^−2^, 0.5 mAh cm^−2^, 5000 h (Li_6_PS_5_Cl solid‐state‐electrolyte)	Coin cells, 83%@1800 cycles, 1 C; (≈2.6 mAh cm^−2^ NCM811, **N/P =** 1.0)	[108]
Li‐Mg‐Sn	Rolling Mg/Sn‐co‐doped Li metal	25≈40 µm (≈3–5mAh cm^−2^	1 mA cm^−2^, 1 mAh cm^−2^, 4000 h (2 M LiFSI in DME/TFTFE)	Multi‐layer pouch 94.2% @140 cycles, 0.2 /0.5 C; (4.4 mAh cm^−2^ NCM811, N/P = 1.1, ≈5 × 8 cm^2^)	[57]

It is worth noting that these interfacial strategies facilitate the rolling process by altering the bulk and surface microstructure of the Li foil. However, a detailed understanding of how the modified foil microstructure evolves during extended cycling is incomplete and represents an excellent opportunity for new science. There is extensive work to be done in quantifying how the initial bulk and surface properties of the modified foil are retained or degraded due to extensive electrochemical cycling. While the modification approaches reported are generally effective in extending cycling life and improving rate capability, are they limited by the retention of these artificial bulk/surface attributes? For example, does the initially introduced artificial F‐rich SEI or C‐rich surface later ultimately degrade? And if so, is that what causes the eventual cell failure, albeit at a higher cycle number than for the unmodified baseline? Otherwise, does the favorable (110) Li metal texture persist after a long cycle? Admittedly, characterizing post‐cycled interfaces is a major challenge, with thick and heterogeneous SEI layers, often with extensive porosity, and electrodeposit morphology that evolves from a dense film to a series of metallic filaments. To this end, cryogenic electron microscopy (cryo‐EM) (including cryogenic focused ion beam cryo‐FIB) and synchrotron imaging are effective in understanding how interfacial modification affects post‐cycled Li electrodeposits, both in modified form and as the unmodified baseline.^[^ ^92^ ^]^

As discussed, a key strategy to facilitate the enhanced mechanical processing of thin Li foils is to introduce alloying elements. These elements form solid‐solution alloys and/or second‐phase intermetallics within the bcc Li matrix, impeding dislocation glide and diffusional dislocation climb. Alloying gives improved creep strength and work‐hardening during rolling deformation, leading to better processability. While the alloying elements need some solubility in Li metal, it may be relatively limited since several weightpercent concentrations are sufficient to promote improved creep strength. There is an extensive body of scientific literature on understanding and improving the room temperature creep strength of Sn‐based solders, many of which operate at comparable homologous temperatures to Li metal (e.g., Sn─Pb eutectic melting point 183 °C).^[^ ^110^, ^111^, ^112^, ^113^ ^]^ These earlier studies may be used as a guide to understanding the efficacy of relatively low alloying additions in improving the rolling processability of Li metal foils. Moreover, creep deformation is likely an important aspect of the Li microstructure during electrochemical cycling, as there are significant chemo‐mechanical effects (electrochemistry, reactivity, stress) involved. Cycling lifetimes and dendrite resistance may both be favored by Li alloys that display improved creep strength. Examining the “old” creep of solder literature may therefore be useful for the microstructural design of Li alloys, with the dual goal of processability and electrochemical stability.

## 3 Metallurgical Casting Methods

### 3.1 Wetting Behavior of Molten Lithium

It is now recognized that the major difficulty of molten Li casting is its wettability onto unmodified copper, which is lithiophobic.^[^ ^114^ ^]^ The various approaches reported to address this problem may be considered from a vantage of the simplified Young's Equation and the various ways that the surface tensions may be tuned. A straightforward force balance at a point does a good job of illustrating the design “knobs” available for tuning the thermal wetting behavior of molten lithium to achieve progressively thinner as‐cast dimensions. The surface tensions balance at the point: γ_ls_+ γ_l_cos θ = γ_s_, where γ_ls_ is the liquid‐substrate surface tension, γ_l_ is the liquid surface tension, and γ_s_ is the substrate surface tension. The units of surface tensions are in energy per unit area, e.g., J m^−2^, which is equivalent to force times distance per unit area, N*m m^−2^, i.e., N/m, which is in turn equivalent to stress times distance i.e., Pa*m. The equilibrium balance of forces resolved parallel to the support (in Young's Equation, forces resolved normal to the support are ignored) establishes the thermodynamic shape of the liquid metal. The wetting angle is defined as cos θ = (γ_s_ − γ_ls_)/ γ_l_. When the inequality γ_s_ − γ_ls_ ≥ γ_l_ is satisfied, a wetting angle of 0° is obtained, while as the inequality γ_s_ − γ_ls_ < γ_l_ increases, the liquid lithium is not perfectly wetted to the support. While wetting angle values of 90° or 65° are often considered as demarcating between wetting and non‐wetting behavior, in practice, an angle of ≈0° would be needed to form thin conformal films of lithium on a given support. Any degree of de‐wetting or local roughness in the films would translate into degraded or uneven electrochemical performance with site‐to‐site variations across the foil.

An additional caveat to implementing Young's Equation to understand the wetting of molten lithium is that a perfectly planar interface is assumed. However, it is possible to employ systematically roughened current collectors, which affect the wetting angle in the following way: Improved or degraded wetting due to increased surface roughness is described by the classic formulation put forth almost ninety years ago by Wenzel.^[^ ^115^, ^116^, ^117^ ^]^ The approach of roughening a hydrophilic surface to make it super‐hydrophilic, or a hydrophobic surface to make it superhydrophobic, is commonly employed in industrial applications. Per Wenzel, a roughness enhancement modifies wetting because of the increased surface area of the liquid–support interface relative to the projected geometric contact area. This affects γ_ls_ and γ_s_, since the true area is larger than the geometric area. It does not affect the liquid surface tension since that interface is with vapor rather than with the support. A dimensionless roughness factor r expresses this enhancement, being the ratio of the true surface area of the interface vs the geometric. The result is a straightforward relation r = cosθ*/cosθ, where θ* is the measured wetting angle on the roughened surface, and θ is the wetting angle established by Young's Equation. The reduced/increased molten lithium wetting angle is θ*, while the wetting angle on planar support with identical chemistry is θ. Since the maximum attainable value of the cosine function is one, the Wenzel Equation is restricted to moderate surface roughness. However, it still gives a good qualitative indication of how geometrical roughness improves liquid wetting. As will be discussed below, the existing approaches toward thinner and more uniform metallurgically cast lithium films involve tuning one or several terms in Young's Equation to promote wetting while in parallel roughening the surface.

Importantly, modeling studies reported that the wetting behavior of molten Li on a Cu current collector is surface chemistry dependent.^[^ ^123^, ^124^, ^125^, ^126^ ^]^ Lithium readily wets a pristine metallic Cu surface, but does not wet a surface terminated by Cu_2_O. Since at ambient conditions Cu metal is covered by a layer of native oxide, this would explain the widely reported lithiophobicity of non‐pretreated foils. Common pretreatment methods for Cu current collectors include plasma cleaning,^[^ ^127^ ^]^ electrochemical reduction,^[^ ^128^ ^]^ and surface modification,^[^ ^129^ ^]^ which would be useful in removing the Cu_2_O or other contaminants on the Cu surface. Of course, the native oxide would readily reform even in a dry room environment.

**Figure 5:**
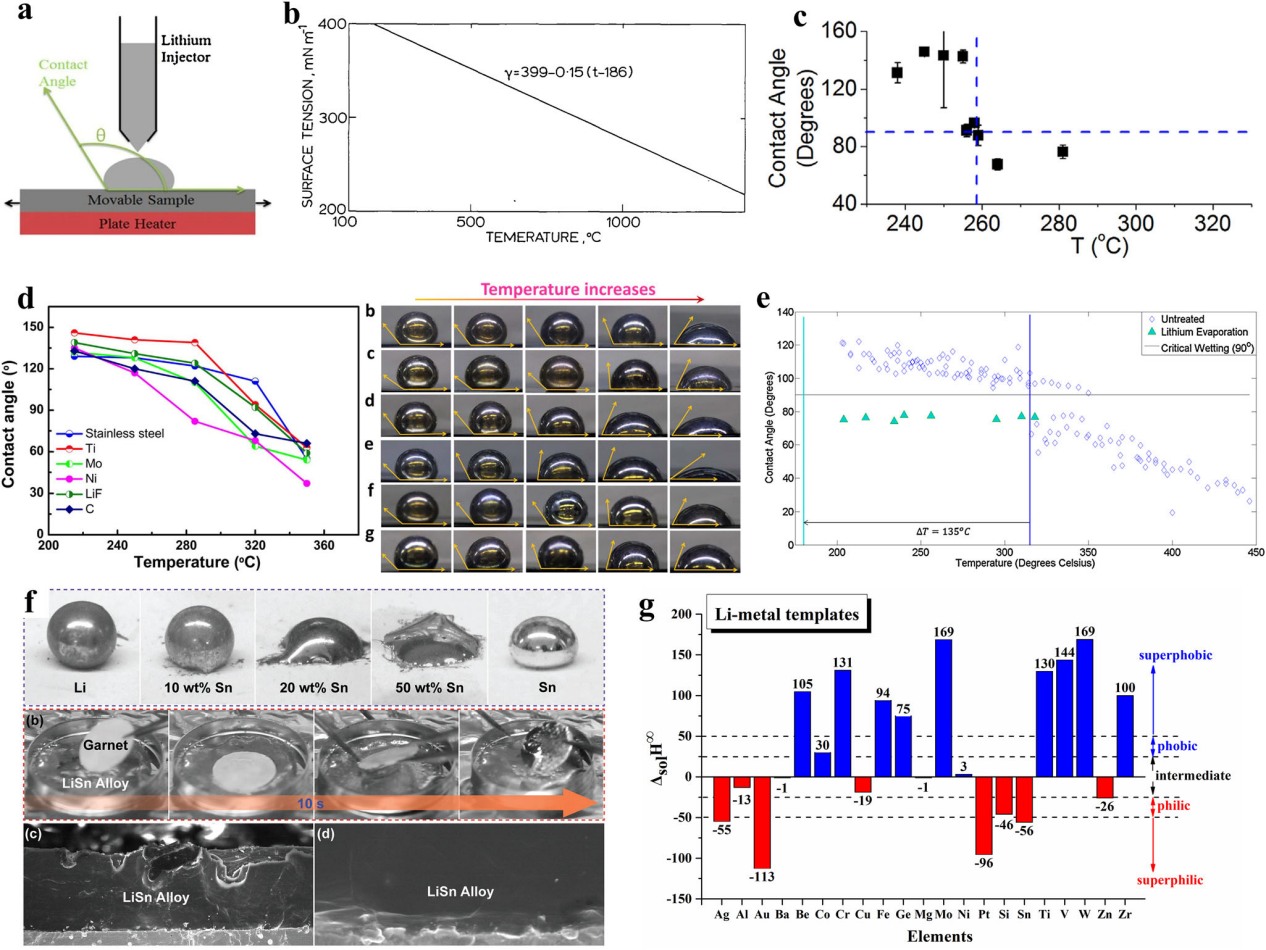
a) General schematic for experimental setup for measuring the wetting behavior of lithium droplets on various supports.^[^ ^118^ ^]^ b) Surface tension of molten Li as a function of temperature, in mN m.^[^ ^119^ ^]^ c) The measured contact angle of molten Li on the lithium oxide surface, with similar wetting behavior obtained on lithium nitride and lithium carbonate.^[^ ^120^ ^]^ d) Analysis of lithium wetting on various supports as a function of temperature and associated photographs for stainless steel foil, Ti foil, Mo foil, Ni foil, and stainless steel coated with LiF and carbon film. The support temperature increases from left to right: 215, 250, 285, 320, and 350 °C. e) The measured contact angle of molten Li on stainless steel, and stainless steel with a micron‐scale layer of evaporated Li as a precursor.^[^ ^121^ ^]^ f) Thermal wetting behavior of Li–Sn alloys on inert supports, top row showing the photographs of the droplets, middle row showing photographs of a garnet disk being dipped into the molten LiSn alloy, bottom row showing cross‐sectional SEM images of the solidified interface.^[^ ^122^ ^]^ g) Tabulated enthalpy of mixing at infinite solution ΔsolH∞ for various elements with lithium, indicating that alloying supports with large negative ΔsolH∞ promote thermal and electrochemical wetting. Panel a reproduced with permission: Copyright 2017, Elsevier. Panel b reproduced with permission: Copyright 1993, Maney Publishing. Panel c reproduced with permission: Copyright 2018, Elsevier. Panels d and e reproduced with permission: Copyright 2014, Elsevier. Panel f reproduced with permission: Copyright 2018, Wiley Panel g reproduced with permission: Copyright 2020, American Chemical Society.^[^ ^78^ ^]^

**Figure** **5**a provides a general schematic for an experimental setup for measuring the wetting behavior of lithium droplets on various supports.^[^ ^118^ ^]^ The techniques for optically measuring the wetting angle are fairly general, being employed for a range of liquids and molten metals. With Li, however, an inert atmosphere must be used since the presence of even trace oxygen or water vapor will skew these results toward a lower wetting angle, since trace concentrations of O_2_/H_2_O are known to reduce the surface tension of molten metals.^[^ ^130^ ^]^ While more sophisticated approaches involve a thermally uniform environment, so as to avoid convection effects due to the hotplate thermal gradient, a conventional unidirectional heat source (hotplate) is normally employed. The equilibrium wetting angle, as well as the rate of spreading, is captured using a conventional optical camera. The molten Li is injected using a thermally heated syringe, while the various planar or roughened supports are laid directly on the hot plate. The thermal conductivity of conventional Cu foils is sufficient to ensure minimal thermal gradients during the experiments. However, this is not guaranteed with supports with extensive porosity that will contain insulating air pockets, such as 3D textured collectors.

Figure 5b provides an experimentally measured plot of the surface tension (γ_l_) of molten Li as a function of temperature, presented in mN m^−1^.^[^ ^119^ ^]^ It may be observed that one straightforward way to promote enhanced wetting of Li on any support is to raise the casting temperature, thereby lowering the surface tension. All things being equal, Li will ball up less with increasing temperature. Moreover, since the viscosity of a liquid decreases with temperature, the melt will be more fluid. Figure 5c illustrates that Li_2_O is highly lithiophobic, with a minimal wetting contact angle(≈60°) being achieved only above 260 °C.^[^ ^120^ ^]^ Similar wetting behavior is reported with molten Li on lithium nitride (Li_3_N) and lithium carbonate (Li_2_CO_3_). These results indicate the importance of Li reaction products in altering wetting, specifically for cases where there is adsorbed water on the current collectors. In that case, the first few layers of Li to come in contact with the Cu (or other collector materials) surface would be oxidized and would favor the subsequently deposited metal to de‐wet. Therefore, a layer of Li_2_O on the collector surface has the effect of increasing the inequality γ_s_ − γ_ls_ < γ_l_, and inhibiting the ability to cast a uniform thin film.

Figure 5d presents lithium wetting angle analysis and associated light optical images as a function of temperature and support types, including stainless steel foil, Ti foil, Mo foil, Ni foil, as well as stainless steel foil coated with LiF, and with a carbon film. The support temperatures for each analysis performed are 215 °C through 350 °C. It may be readily observed that for all support types, the wetting angle decreases with increasing temperature. This is expected due to the lower surface tension of molten Li. Moreover, the amount of wetting improvement with temperature appears to be consistent with all supports, the Ni support 280 °C data being perhaps the only notable outlier. This substantiates the argument that the wetting improvement is liquid surface tension driven, rather than due to an underlying change in γ_ls_ or γ_s_. The role of interfacial reactivity in modifying the lithium–support interface tension γ_ls_, will be discussed later. The standard enthalpy of the formation of NiO_2_ is −240 kJ mol^−1^, making it readily reduced by metallic Li. The standard enthalpy of formation of TiO_2_ is −940 kJ mol^−1^, making it relatively stable in contact with Li. The enthalpy of formation of MoO_3_ and LiF are −745.17 and −616.9 kJ mol^−1^, making both relatively stable against metallic Li. As discussed in the previous section, both Cr_2_O_3_ and Cr_3_O_4_ are stable against Li metal. There is negligible bulk solubility of Li in any of these metals. Carbon supports are nominally inert against Li, although there may be some insertion of Li atoms into the bulk structure and/or bonding with the oxygen and hydroxide groups on the surface. Therefore, these nominally inert supports display a relatively consistent pattern of Li wetting angle vs temperature and may be used as a baseline to understand more reactive systems discussed next.

Figure 5e presents the measured contact angle of molten Li on stainless steel and on stainless steel with a micron‐scale layer of evaporated Li as a seed layer. While nearly complete wetting is not achieved until above 400 °C, it may be observed that at low temperatures, the Li seeding layer reduces the wetting angle by over 40°. However, wetting is still poor, with a wetting angle of ≈80°.^[^ ^121^ ^]^ This demonstrates that thermal wetting of molten Li on pre‐existing Li is relatively poor, at least for the experimental setup employed. This is despite the authors’ assertion that the Li seeding layers did not de‐wet the support once physically vapor deposited, even at temperatures of 200 °C to over 300 °C. Figure 5f demonstrates the thermal wetting behavior of Li–Sn alloys on inert supports.^[^ ^122^ ^]^ The top row displays photographs of the Li droplets with various Sn content. The middle row displays photographs of a garnet disk being dipped into the molten Li‐50%Sn alloy, the composition corresponding to the optimum wetting. The bottom row displays cross‐sectional SEM images of the solidified interface. The observed wetting behavior demonstrates how alloying can promote uniform wetting of Li. The underlying alumina or garnet supports should not be reactive with Li or Sn, indicating that alloying changes the surface tension (γ_l_) of the molten Li.

Liu and Mitlin proposed that the enthalpy of mixing at an infinite solution (Δ_sol_H^∞^) can be used to predict the wetting behavior of Li on a given support.^[^ ^131^ ^]^ The values of Δ_sol_H^∞^ are a direct indicator of the thermodynamic affinity between the Li atoms and the support atoms. Figure 5g presents the tabulated enthalpy of mixing at infinite solution Δ_sol_H^∞^ for various elements with lithium, indicating that alloying supports with large negative Δ_sol_H^∞^ promote thermal and electrochemical wetting. Higher interfacial solubility of the support is therefore directly correlated with its improved lithiophilicity. The figure shows the Δ_sol_H^∞^ values of Li dissolving into varying elements, where one may see that the Δ_sol_H^∞^ of Au,^[^ ^132^ ^]^ Ag,^[^ ^133^ ^]^ Pt,^[^ ^134^ ^]^ Si,^[^ ^114^ ^]^ Sn,^[^ ^135^ ^]^ Zn^[^ ^136^ ^]^ are negative. This agrees with numerous experimental studies where the main constituent of the lithiophilic host is based on Au, Ag, Pt, Sn, or Zn. The elements Si and Al would also be effective due to the negative enthalpy. However, both are covered by a passivating surface oxide that is not readily reduced, with standard enthalpy of formation of SiO_2_ and Al_2_O_3_ being −911 and −1676 kJ mol^−1^. While Li reversibly alloys with Si and with Al, the stable native oxides may initially impede rapid wetting on their surfaces. In addition, the electrochemical alloying of Al and Si by Li is known to be kinetically sluggish. Both Si and Al chemically react with Cu to form highly stable binary intermetallics (Al_2_Cu, Cu_15_Si_4_, others), with the wetting behavior of Li being likely altered as a result. Combined, these factors may make Si and Al less useful as coatings for Cu supports. Per Figure 5g metallic Cu is also expected to be lithiophilic. However, as discussed previously, the native oxide present on its surface would render it lithiophobic.

Another method to promote enhanced thermal and electrochemical wetting of Li onto support is to employ lithiophilic intermetallics, such as Li_22_Sn_5,_^[^ ^137^ ^]^ Li_9_Al_4_,^[^ ^138^ ^]^ Li_15_Si_4_,^[^ ^139^ ^]^ and Li_13_In_3._^[^ ^140^ ^]^ For example, atomic simulations combined with experiments have confirmed that *fcc* antifluoride Li_2_Te archetype intermetallics are lithiophilic and promote the wetting of Li on their surfaces.^[^ ^141^ ^]^ For both interfacial alloying and interfacial intermetallic formation, the relevant Young's Equation parameter that is modified is the molten lithium–support surface tension (γ_ls_). The formation of alloying layers or intermetallic layers at the lithium–support interface would reduce the value of γ_ls_ and promote wetting vs a thermodynamically inert interface. The reaction between the surface oxygen and hydroxide groups of the carbon support and the molten Li metal would have a similar effect, promoting wetting over an inert carbon surface. The intercalation of Li into a material such as graphite or graphene would likewise promote lithiophilicity by reducing the value of γ_ls_.^[^ ^9^ ^]^

### 3.2 Chemically and Physically Tuned Lithiophilicity

**Figure 6:**
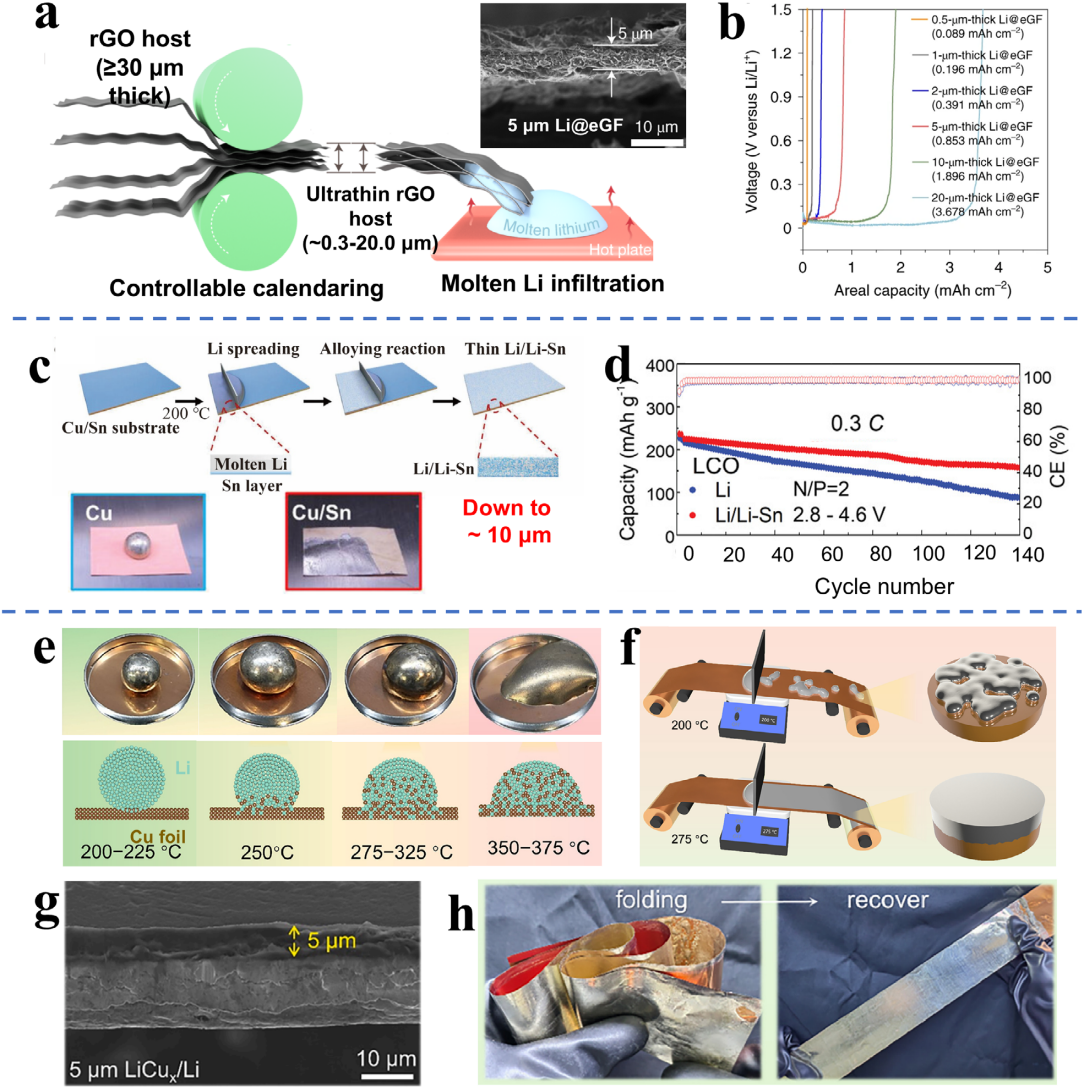
a) Schematic of free‐standing Li@eGF composites film prepared by infiltrating molten Li into rolled thin graphene film, with an inset showing an SEM image of the resultant material b) Strippable Li capacity of Li@eGF film with varying thickness.^[^ ^142^ ^]^ c) A schematic of modifying the Cu surface with lithiophilic Sn to facilitate the spreading of molten Li, the bottom row presents photographs of the wetted alloy layer vs unwetted pure Li baseline, d) Cycling performance of Li||LCO full cells at 0.3C and 2.8–4.6V, adopting 20 µm Li/Li‐Sn vs pure Li.^[^ ^143^ ^]^ e) Wettability of molten Li droplet and Cu foil as a function of temperature.^[^ ^144^ ^]^ The top row shows photographs of the melt between 200 and 375 °C, bottom row provides schematics of the proposed wetting enhancement mechanism based on mutual solubility. f) Promoting the wetting of Li onto Cu at elevated temperatures. g) SEM image of the post‐solidified (275 °C) Li layer on the Cu support. h) Photographs of the produced LiCu foil. Panels a and b reproduced with permission: Copyright 2021, Springer Nature. Panels c and d reproduced with permission: Copyright 2021, Wiley. Panels c to h reproduced with permission: Copyright 2023, Elsevier.

Cui's group prepared a reduced graphene oxide sheet and then calendared it into a thin film format with a controllable thickness of 0.5–20 µm. After introducing the molten Li into its interlayer space, a free‐standing Li‐graphene composite foil (Li@eGF) can be fabricated.^[^ ^142^ ^]^ Those results are presented in **Figure** **6**a. The 2–5 µm Li@eGF foil was compressed onto the Si‐electrodes to serve as a Li‐source for pre‐lithiation. Pre‐lithiated Si anodes achieved an initial coulombic efficiency (CE) of approaching 100%. As shown in Figure 6b, the capacity of Li@eGF foil is directly correlated and tunable with its thickness. Per Figure 6c, Sun's group introduced a lithiophilic Sn nanolayer (≈0.2 mg cm^−2^) onto Cu foil by magnetron sputtering.^[^ ^143^ ^]^ This lithiophilic Sn layer facilitated the wetting of thin molten Li (10–50 µm) by doctor‐blade casting processing, due to the in situ formation of the Li‐Sn alloy interlayer. The obtained Li/Li‐Sn alloy foil (20 µm) showed capacity retention of 77% after 100 cycles at 0.3C in an LCO full cell, with a cathode areal capacity of 2.8 mAh cm^−2^ and an N/P ratio of 2. Those results are shown in Figure 6d.

Li's group reported that the wetting of molten Li and Cu foil could be significantly improved with increasing temperature.^[^ ^144^ ^]^ Those results are presented in Figure 6e,f. The authors attributed this effect to enhanced mutual solubility of Cu and Li at elevated temperatures, although reduced surface tension of Li due to higher temperature would also explain the improvement. A wetted Li layer with a thickness of 5–50 µm was successfully prepared on a Cu, as shown in Figure 6g, waiving the need for a lubricant or support film. The molten Li was poured on the heated Cu foil and then coated using a tailor‐made spreader. After that, the adding rate of Li liquid and the rolling speed of Cu foil were coordinated to realize the continuous production of LiCu/Li composite film, as highlighted in Figure 6h.

**Figure 7:**
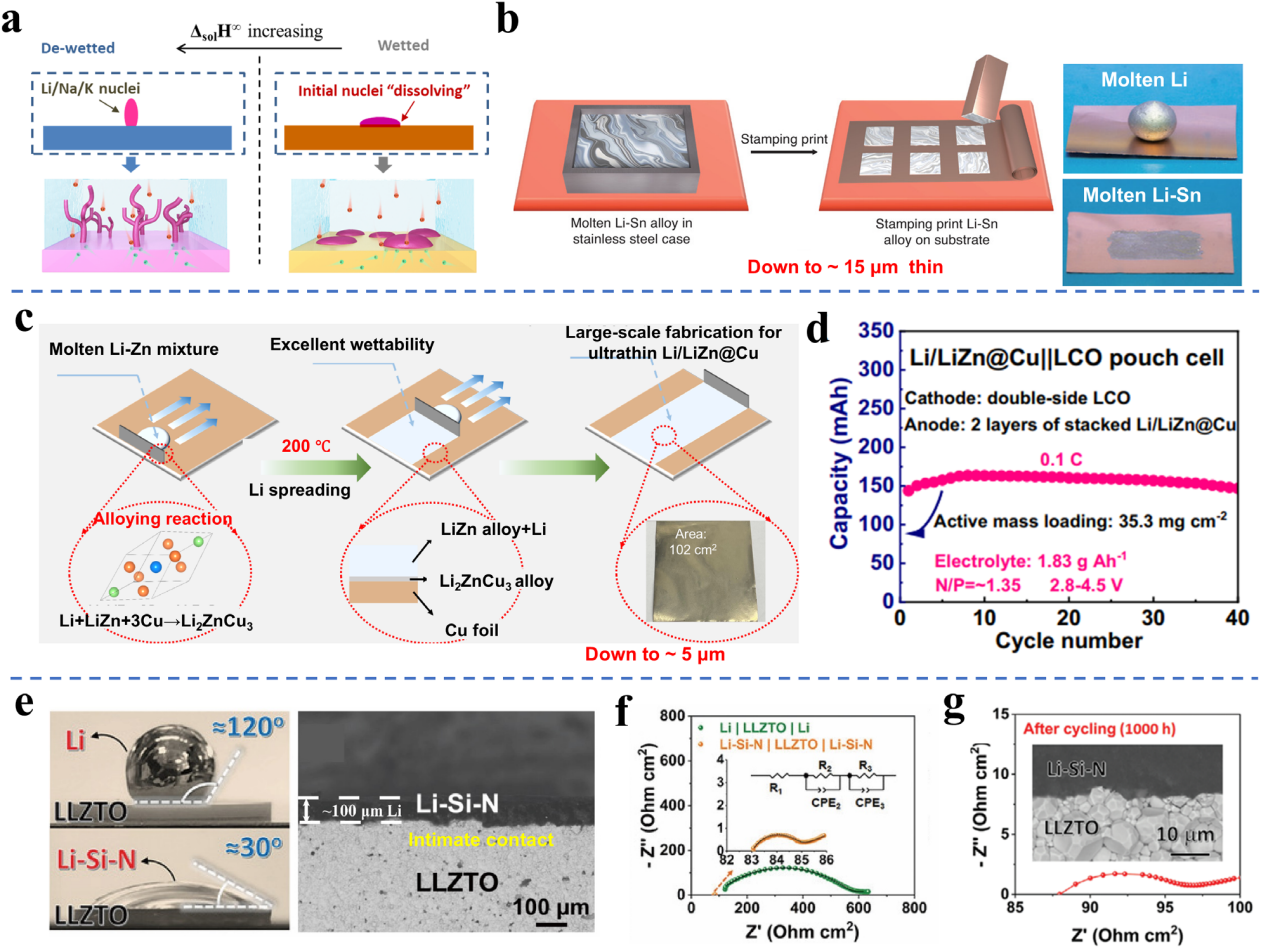
a) Schematic illustration of the concept of Li dissolving into various elemental support materials with positive or negative enthalpy of mixing of infinite solution (Δ_sol_H^∞^), where positive Δ_sol_H^∞^ points to limited alloying with promoted electrochemical de‐wetting that ultimately leads to filament‐like dendrites.^[^ ^131^ ^]^ b) Schematic of molten Li–Sn alloy (5 wt.% Sn) wetting with Cu foil that allows direct stamping print.^[^ ^145^ ^]^ c) Schematics of direct coating of molten Li–Zn (20 wt.% Zn) onto Cu foils, forming a Li/LiZn@Cu electrode with Li_2_ZnCu_3_ interlayer, d) Cycle performances of pouch cells adopting Li/LiZn@Cu.^[^ ^56^ ^]^ e) Contact angle of molten Li and Li with 1 wt.% Si_3_N_4_ against LLZTO. f) Impedances of all‐solid‐state symmetric cells employing Si_3_N_4_ modified Li and pure Li electrodes, g) SEM images of post‐cycled interface between Si_3_N_4_ modified Li and LLZO. Panel a reproduced with permission: Copyright 2020, The Royal Society of Chemistry. Panel b reproduced with permission: Copyright 2021, Wiley. Panels c and d reproduced with permission: Copyright 2024, Springer Nature (open access). Panels e to g reproduced with permission: Copyright 2021, Wiley.^[^ ^146^ ^]^

As illustrated by the schematic in **Figure** **7**a, tuned surface thermodynamics are effective in promoting uniform wetting and uniform dewetting of the molten Li metal. Molten Li infusion into a porous host with a lithiophilic skeleton represents an effective methodology for extending the cycling life of LMBs.^[^ ^147^, ^148^ ^]^ This takes advantage of the wetting energetics and the Wenzel surface area enhancement effect. Zheng's group fabricated a ≈350 µm thick Janus textile host comprising a lithiophobic Ni side and a lithiophilic NiSb side.^[^ ^149^ ^]^ The molten Li was induced to selectively wet the NiSb side, forming a 50 µm layer of Li. Due to the lithiophobicity of the Ni side, Li prefers to deposit into the bottom side of the porous textile framework. Such a judicious design of a porous host can also mitigate the growth of Li dendrites that would occur during extended cycling.

Adjusting the composition of the melt represents another approach for achieving thin and uniform Li films onto collector supports. The strategy is again to modify Young's Equation to achieve enhanced thermal wetting. Hu et al. prepared a molten Li‐Sn alloy (5 wt.% Sn) and then directly stamped the melt onto a Cu substrate at 200 °C, forming a ≈15 µm Li─Sn alloy layer with a controllable shape.^[^ ^145^ ^]^ This methodology is illustrated in Figure 7b. The introduction of Sn greatly enhances the melt's wettability with Cu, forming a 15 µm Li–Sn alloy layer with strong adhesive bonding to Cu foil. This is likely due to the formation of intermediate Cu‐Sn intermetallics at the metal–collector interface. As shown in Figure 7c, implementing such a 15 µm thin alloy anode, supported by Cu foil, into an NCM‐based pouch cell, enabled stable cycle performance. Lu's group reported a Li─Zn alloy melt (mass ratio Li:Zn = 4:1) that displayed improved wettability on Cu, allowing it to be uniformly spread into large areas by doctor‐blade casting.^[^ ^56^ ^]^ The wettability was attributed to the lower surface tension of the molten Li‐Zn alloy as compared to pure Li melt, and the interfacial reaction by in situ forming Li_2_ZnCu_3_. An ultrathin layer of Li/LiZn was successfully coated onto Cu foil, yielding a thin Li/LiZn@Cu anode that promoted stable electrodeposition/dissolution. Ultimately, a high mass loading LCO‐cathode‐based pouch cell achieved 40 stable cycles under N/P ratio (1.35) and lean electrolyte (1.83 g Ah^−1^), as shown in Figure 7d.

Oxide‐based solid‐state electrolytes (SSEs), such as garnet‐type lithium lanthanum zirconium oxide (LLZO) and lithium lanthanum zirconium tantalum oxide (LLZTO), are relatively inert. Being incompletely wetted by molten Li leads to high interfacial impedance due to poor contact of the solidified metal and the electrolyte. Introducing a ≈5 µm porous Zn layer (PZL) onto SSE by magnetron sputtering was shown to be highly effective in improving this issue.^[^ ^150^ ^]^ The molten Li reacted with the Zn, resulting in a 10 µm thick alloy layer, which reduced the LLZTO/Li interfacial impedance during cycling. Studies have found that the molten Li–Mg alloy (20 wt.% Mg) showed a small contact angle with LLZO garnet‐type SSEs.^[^ ^105^ ^]^ Shao et al. added 1 wt.% Si_3_N_4_ to molten Li at 250 °C, promoting its thermal wetting on the LLZTO.^[^ ^146^ ^]^ As shown in Figure 7e, the contact angle decreased from 120° to 30°. The symmetric cells adopting 100 µm Li‐Si‐N and LLZTO show much‐reduced interface resistance as compared to the neat Li counterparts surviving 1000 cycles. Those results are illustrated in Figure 7f,g. To provide a direct comparison of the thin Li anodes prepared through metallurgical casting, we condense the reported data into **Table** **2**.

**Table 2:** Summary of electrochemical performances of thin Li metal anodes by metallurgical casting.

Materials type	Processing method	Electrochemical performances			Refs.
		Thickness (area capacity)	Half‐cell performances (electrolytes)	Full cell performance (cathode, N/P ratio, size)	
Li_x_Cu/Li	Spreading molten Li onto Cu foil at a high temperature	5–50 µm (1–10 mAh cm^−2^)	1 mA cm^−2^, 1 mAh cm^−2^, 1200 h (1 M LiTFSI in DME/DOL 1:1 vol + 2% LiNO_3_)	Coin cells ≈100%@220cycles,1C; (≈2.45 mAh cm^−2^ LFP, **N/P =** 1.6)	[144]
Li@Cu_2_O/Cu	Spreading molten Li onto Cu_2_O/Cu	1–30 µm (0.2–6 mAh cm^−2^)	0.1 mA cm^−2^, 0.1 mAh cm^−2^, 100 h (1 M LiPF_6_ in EC/DME (1:1))	Coin cells ≈81%@350cycles,0.2C; (≈1 mAh cm^−2^ NCM 9055, **N/P =** 1)	[151]
Janus Li anode	Infusing molten Li into Janus textile	50 µm (10 mAh cm^−2^)	10.0 mA cm^−2^,1 mAh cm^−2^, 1000 h (1 M LiTFSI in DME/DOL 1:1 vol + 2% LiNO_3_)	Single‐layer pouch cells, ≈100%@150 cycles; (≈1.8 mAh cm^−2^ LCO, **N/P =** ≈**5.0**, **N/A**)	[149]
Li/LiZn@Cu	Spreading molten Li‐Zn alloy onto Cu foil	5–48 µm (0.9–9 mAh cm^−2^)	1.0 mA cm^−2^, 1 mAh cm^−2^, 700 h (1 M LiTFSI in DME/DOL 1:1 vol + 1% LiNO_3_)	Multi‐layer pouch cells ≈100%@40cycles,0.1C; (≈3.3 mAh cm^−2^ LCO, **N/P =** ≈1.3, ≈5 × 5 cm)	[56]
Li/Li‐Sn@Cu	Spreading molten Li on Sn‐coated Cu foil	10–50 µm (2≈10 mAh cm^−2^)	1.0 mA cm^−2^, 1 mAh cm^−2^, 900 h (1 M LiTFSI in DME/DOL 1:1 vol + 2% LiNO_3_)	Coin cells 77%@140cycles, 0.3 C; (≈2.7 mAh cm^−2^ LCO, **N/P = 2.0, N/A**)	[143]
Li‐Sn@ Cu	Stamping molten Li on Cu substrate	15 µm (≈3 mAh cm^−2^)	0.25 mA cm^−2^, 0.04 mAh cm^−2^, 100 h (1M LiPF_6_ in EC: DMC (1:1))	Single‐layer pouch cells 97.5% @ 25 cycle, 0.1 C (0.9 mAh cm^−2^, NCM 523, **N/P = 3.3, N/A**)	[145]
Li‐Si_3_N_4_	Spreading molten Li on LLZTO SSE	100 µm (≈20 mAh cm^−2^)	0.4 mA cm^−2^, 0.4 mAh cm^−2^, 1000 h (LLZTO|PEO solid‐state electrolyte)	Coin cells ≈97%@100 cycles, 1 C (≈0.3 mAh cm^−2^, LFP, **N/P = 66.7, N/A**)	[146]
Li‐Zn	Spreading molten Li on Zn‐coated LLZTO SSE	25 µm (≈5.15 mAh cm^−2^)	0.1 mA cm^−2^, 0.05 mAh cm^−2^, 1000 h (LLZTO solid‐state electrolyte)	Coin cells 100%@170cycles, 0.1C (≈0.8 mAh cm^−2^, NCM 523, **N/P = 6.4, N/A**)	[150]

At a high level, the interrelations between the support wettability and the effectiveness of the solidification casting process need to be further examined. It is recognized that support wettability significantly impacts the onset of unstable morphological evolution during electrodeposition and electrodissolution of lithium metal anodes. However, the role of wettability in the achievable film thickness, the as‐solidified film morphology, its bulk microstructure (grain size, texture, porosity, *etc.)*, and even its surface chemistry (e.g., rougher film surfaces may be more reactive) needs to be better understood. Intuitively, one expects that “better” substrate wetting by molten lithium leads to “better” films. However, is it possible to actually quantify these effects, e.g., directly correlating achievable film thickness and/or roughness with support alloying behavior? What is the role of chemical reactivity and the associated chemo‐mechanical stresses during solidification? Can this be quantified in terms of the resultant bulk and surface properties listed above? How does reactivity during solidification affect film grain size, texture, roughness, etc., and how do these “initial” properties translate into changes in the electrochemical stability of the anode?

During solidification, poor wettability leads to uneven Li nucleation, leading to agglomerated morphologies with reduced connectivity and limited electrochemical utilization of the substrate. These localized deposits are high‐energy sites prone to the initiation of interface instabilities such as dendrite growth. With respect to the electro‐dissolution process, poor wetting during solidification may lead to incomplete dissolution, resulting in “dead Li” or voids. Such linkages between the support properties, the solidified electrodes, and the resultant electrochemical behavior require further analysis. Ideally, model support systems should be employed (e.g., uniform Ag, Au, or Mg layers), such that the results are repeatable from group to group. The interplay between substrate wettability during the casting fabrication and the ultimate electrochemical performance of LMBs requires further analysis. This includes the role of initial support properties not only in cycling life but also in rate capability and critical current density. What is also (generally) unknown is how much/whether, lithiophilicity of the support during solidification affects the intrinsic SEI structure/chemistry. How do the support – deposit energetics during casting affect the electrodeposit – ambient environment and electrodeposit – electrolyte reactivity?

## 4 Solution‐Based Wet Methods

### 4.1 Slurry Coating of Lithium Powders

Thin Li films are also produced by employing lithium precursors that are dispersed in a liquid medium, i.e., solution‐based wet methods. A major benefit of such wet methods is that they can be performed at ambient temperature and atmosphere. Normally, two types of liquified precursors could be used, based on “powdered lithium” and “ionized lithium.” For example, stabilized Li metal powders (LMPs) are employed as active particles for thin LMA fabrication. The process is akin to mainstream electrode preparation, involving particles of the active material in a suspension, which is cast onto a current collector. After removing the solvent with thorough drying, the electrode is calendered into a thin Li metal active layer with the targeted thickness. The synthesis of LMP relies on a droplet emulsion technique, where the molten Li is mixed with silicone oil at an elevated temperature to form the emulsion. The solidified LMPs are obtained by cooling the emulsion to room temperature, followed by further purification.^[^ ^152^ ^]^ However, the highly reactive nature of LMP poses potential safety risks during production, even when handled in dry‐air conditions. To mitigate this, protection layers based on Li_2_CO_3,_ LiF, or organo‐wax are formed by introducing additives into the emulsion.^[^ ^153^, ^154^ ^]^ Despite the success of the protection layer approach, attaining targeted electrochemical performance remains a challenge.^[^ ^154^, ^155^ ^]^

**Figure 8:**
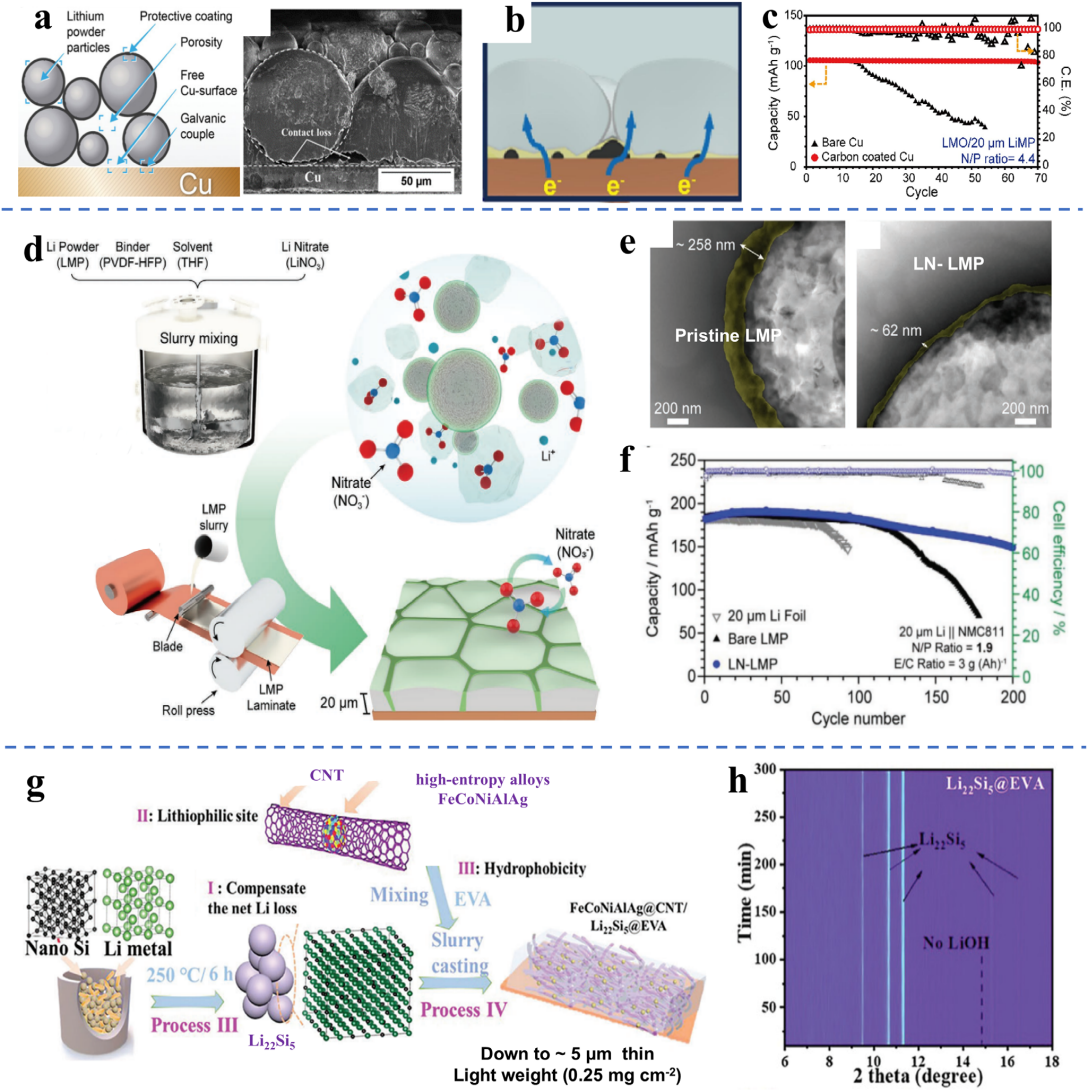
a) Illustration and SEM images of a Li‐metal powder (LMP) coating layer on Cu, being susceptible to galvanic corrosion and loss of interfacial contact.^[^ ^156^ ^]^ b) Schematic illustrating how an introduced carbon interlayer on Cu foil enhances the adhesion of LMP and mitigates galvanic corrosion. c) Li||LMO pouch cell performance employing carbon‐coated Cu as support for LMP.^[^ ^159^ ^]^ d) Schematics of mixing LiNO_3_(LN) to the LMP slurry to produce 20 µm LN‐LMP electrodes, e) TEM images of the thinner passivation layer on LN‐LMP as opposed to LPM, f) Full cell cycle performances of baseline LMP and LN‐modified LMP anodes.^[^ ^154^ ^]^ g) Schematic illustrating a slurry based on high‐entropy alloys, carbon nanotubes, EVA polymer, and Li_22_Si_5_ powder. This slurry is coated onto Cu to fabricate a thin Li‐composite foil. h) XRD analysis of the Li‐composite foil of pouch cells that had been exposed to moisture. Panel a reproduced with permission: Copyright 2020, Wiley (open access). Panels b and c reproduced with permission: Copyright 2021, Elsevier. Panels d to f reproduced with permission: Copyright 2021, Wiley. Panels g and h reproduced with permission: Copyright 2024, Wiley.^[^ ^160^ ^]^

Large area thin (< 20 µm) LMA can be fabricated through the slurry coating, potentially being directly compatible with existing electrode manufacturing processes. Compared to ingot or melt‐based fabrication, LMP‐based LMAs possess a much higher surface area and electrode porosity. This will impact electrochemical cycling in multiple ways. On one hand, the higher surface area will reduce the local current density, allowing for faster charging and potentially higher achievable areal capacity. On the other hand, the higher surface area will cause more SEI formation, leading to accelerated impedance rise and active Li loss. Due to the infiltration of liquid electrolytes into the pores, galvanic corrosion between LMP and the Cu collector may also be an issue. Stan et al. have shown that galvanic corrosion is responsible for the loss of electric contact and poor adhesion.^[^ ^156^ ^]^ This resulted in low active material utilization and declined battery storage life, as shown in **Figure** **8**a.

Employing polymer binders and conductive agents in the LMP slurry has shown progress in alleviating the adhesion and electric contact loss issues that can exist for LMP‐based anodes.^[^ ^157^, ^158^ ^]^ Lee et al. have demonstrated that a carbon layer coating ≈600 nm thick) on the surface of Cu foil can greatly enhance the adhesion of the LMP particles at the as‐prepared state, and throughout cell operation.^[^ ^159^ ^]^ Those results are illustrated in Figure 8b. In parallel, this carbon interlayer suppressed corrosion between LMP and Cu foil, preventing delamination of the LMP from the current collector. Full cells adopting a 40 µm LMP anode and a LiMn_2_O_4_ cathode exhibited capacity retention of 96.4% over 250 cycles (N/P ratio of 8.8). However, when the thickness of the LMP anode was further decreased to 20 µm (N:P = 4.4) and the electrolyte quantity was reduced to 6.1 g Ah^−1^, full cells utilizing a bare Cu‐supported LMP anode ceased to function after 10 cycles. In contrast, with a carbon‐coated Cu support, the full cells demonstrated significantly improved lifespan, achieving 70 cycles, as shown in Figure 8c.

Incorporating Li‐based alloys, instead of pure Li, into the LMP appears to create enhanced electrochemical performance, including enhancing the slurry's air stability. In an early study on the subject, Li‐Si alloy nanoparticles and graphene sheets were incorporated into the slurry, with pure Li particles serving as the baseline. Coating such a slurry onto Cu yielded air‐stable and freestanding Li composite films with a thickness of ≈20 µm.^[^ ^161^ ^]^ As illustrated in Figure 8d, Lee et al. fabricated a 20 µm LMA by adding LiNO_3_ to the LMP slurry.^[^ ^154^ ^]^ The addition of LiNO_3_ triggered chemical nitration of the LMP, enriching its surface with a layer of Li_3_N and LiN_x_O_y_. This layer was significantly thinner than a Li_2_CO_3_/Li_2_O layer on conventional LMP electrodes, per Figure 8e. Uniform surface passivation of LMP particles, in combination with the release of LiNO_3_ into the electrolyte, gave rise to the stable operation of Li || NCM811 full cells over 200 cycles (low N/P = 1.9 and lean electrolytes of 3 g Ah^−1^). This electrochemical performance is presented in Figure 8f. Ma's group developed an ultra‐thin (5 µm) and lightweight (0.25 mg cm^−2^) Li‐composite layer on Cu foil, as illustrated in Figure 8g.^[^ ^160^ ^]^ This layer was composed of Li_22_Si_5_ particles, high‐entropy alloys (HEA) with carbon nanotubes (CNTs), and hydrophobic ethylene‐vinyl acetate copolymer (EVA). In situ XRD revealed that the introduction of EVA is key in suppressing humidity‐related corrosion of Li_22_Si_5_ (forming LiOH), per Figure 8h. A corrosion‐resistant LMA was implemented in a pouch cell with minimal excess Li (N/P<0.1), displaying capacity retention of 90.3% over 100 cycles. The capacity of the control group employing composite foil but without HEA and CNT decayed to 74.6% over 100 cycles.

### 4.2 Electrochemical Deposition of Lithium Films

As lithium metal powders are often in the size of tens of microns, this limits the lower thickness of the resultant LMA. Conversely, electrodeposition from Li salt solution offers improved precision while allowing sub‐micrometer film thickness. Electrodeposition of metal films has been widely implemented in varying industry sectors since the 19th century. A typical electrodeposition device includes two working electrodes immersed in an electrolyte containing the depositing metal ions and an external electrical power supply. The metal film grows on top of the negative electrode at a reductive potential. Theoretically, the production of LMA onto a conductive substrate can be precisely regulated by controlling the current and the passing charges. However, unlike many other metals, producing ideal thin Li layers by electrodeposition is hindered by the extremely high reactivity of Li with H_2_O and many other protic species. This makes the electrodeposition of Li^+^ heavily influenced by the formation of a surface passivating SEI layer. As discussed earlier, non‐uniform growth of Li films is prone to occur and may be mitigated by employing electrically conductive lithiophilic coatings.^[^ ^40^, ^131^ ^]^ The electrodeposited  Li can form the preferred crystallographic texture, which is influenced by the electrochemical deposition parameters, including the electrolyte formula, current density, external pressure, and starting texture of the substrate.^[^ ^41^, ^88^, ^166^, ^167^ ^]^ Roughened electrodeposit promotes the growth of Li during electrochemical cycling.^[^ ^163^, ^164^ ^]^ Slower Li‐deposition rates tend to favor a flat, uniform, and dense structure, at the cost of compromising efficiency and cost for LMA processing.

**Figure 9:**
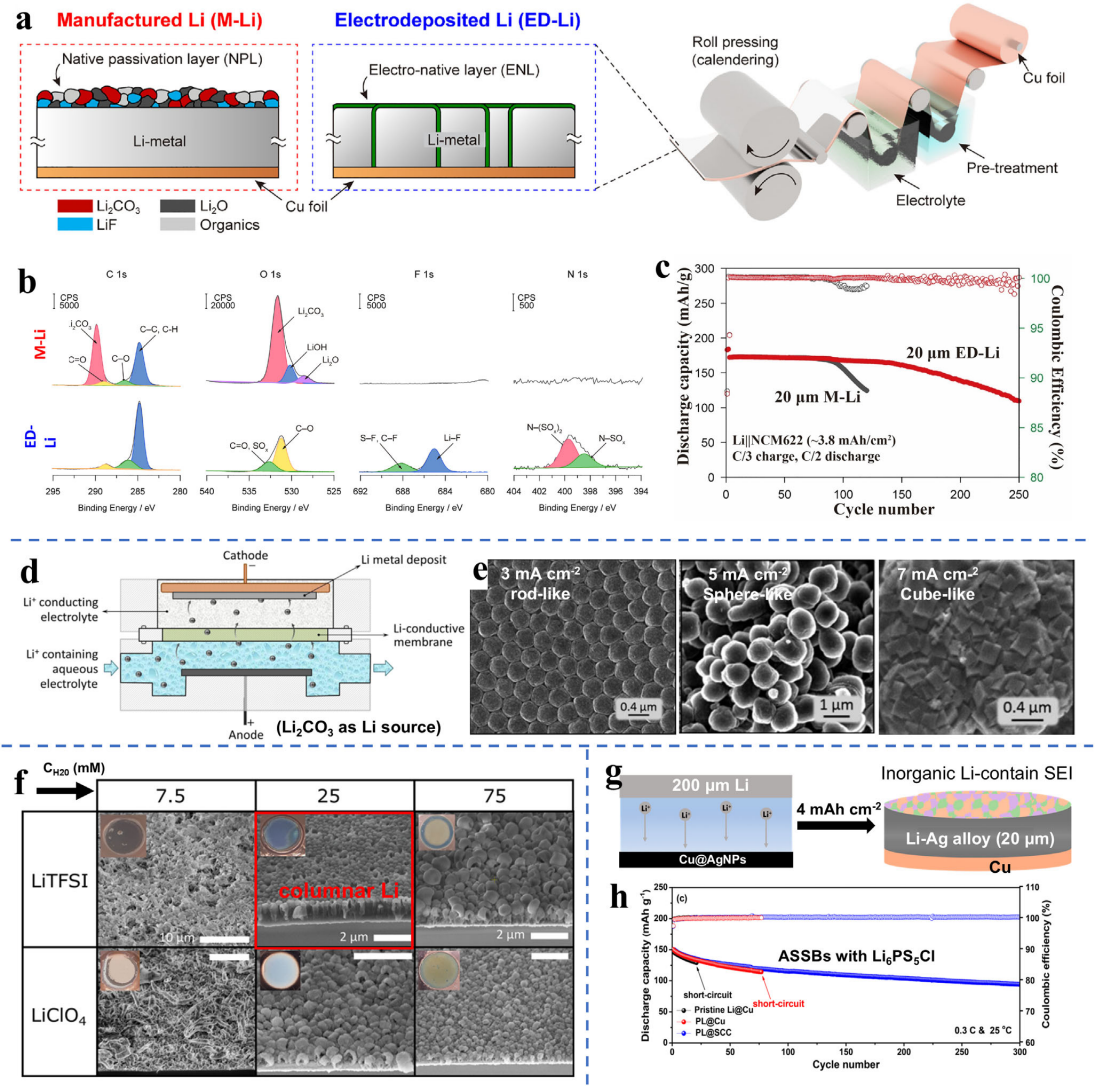
a) Schematics illustrating rolling‐manufactured lithium (M‐Li) and electrodeposited lithium (ED‐Li), both 20 µm in thickness, yet exhibiting differing surface passivation layers (NPL). b) XPS of ED‐Li and M‐Li after disassembling cells, c) Full cell performances employing ED‐Li and M‐Li.^[^ ^162^ ^]^ d) Structural design of the electrolysis of Li_2_CO_3_ to deposit 5 µm high‐purity Li through a Li‐conductive membrane, e) SEM images of Li‐morphologies under varying electroplated current densities.^[^ ^163^ ^]^ f) Li‐deposit morphologies under varying trace amounts of water in the LiTFSI and LIClO_4_‐based electrolytes.^[^ ^164^ ^]^ g) Schematic of silver‐coated Cu (SCC) to enable uniform electrodeposition of a 20 µm Li−Ag layer, h) Cycling performances of all‐solid‐state batteries employing pre‐lithiated SCC as anode. Panels a to c reproduced with permission: Copyright 2023, Elsevier. Panels d and e, reproduced with permission: Copyright 2018, American Chemical Society (open acess). Panel f reproduced with permission: Copyright 2024, American Chemical Society. Panel g and h reproduced with permission: Copyright 2024, American Chemical Society.^[^ ^165^ ^]^

A specially designed electrolyte system may be the key to obtaining the ideal Li solvation and SEI structure. Conventional electrolytes with carbonate solvents, those widely used in Li‐ion batteries, are known to form unstable SEI and promote Li dendrites. Seo et al. reported an ether‐based localized highly concentrated electrolyte (LHCE), promising electrolytes known to have superior compatibility with Li metal anodes, to electroplate 20 µm (≈4.13 mAh cm^−2^) of metallic Li on Cu foil.^[^ ^162^ ^]^ Those results are illustrated in **Figure** **9**a. The electrodeposited Li (ED‐Li) showed uniform and dense morphology, forming an LHCE‐derived native passivation layer uniformly enriched with inorganic species, per Figure 9b. As compared to rolling‐based manufactured Li (M‐Li), that of ED‐Li appears much thinner and flatter. Paired with a high‐loading NCM622 cathode (3.8 mAh cm^−2^, N/P = 1.09), the ED‐Li‐based full cells retained 80% capacity after 200 cycles, while the M‐Li baseline exhibited fast capacity decay after 100 cycles. Those results are illustrated in Figure 9c.

Most existing efforts for electrodeposition adopt expensive Li‐salts (LiPF_6_, LiFSI) as the electrolyte medium and metallic Li as the counter electrode, while a lower‐cost alternative would be very useful. O'Brien et al. reported an electrodeposition system with a circulating aqueous solution containing Li_2_CO_3_ as the Li source and a LiPF_6_‐based electrolyte to deposit 5–30 µm Li film onto Cu substrate (Figure 9d).^[^ ^163^ ^]^ A ceramic membrane was implemented in between, allowing Li^+^ flux but separating aqueous and aprotic solutions. This design allows the use of Li salt instead of metallic lithium as feedstock and may be highly beneficial for commercial applications. With this setup, the authors have demonstrated that the morphology and uniformity of lithium metal films are strongly affected by the applied current density (Figure 9e). The electrolyte formulation is a decisive factor in determining the uniformity of lithium deposition; any impurities in the electrolytes can strongly affect the electrodeposition process due to the high reactivity of metallic Li and the vulnerability of SEI.^[^ ^168^, ^169^ ^]^ As shown in Figure 9f, Vereecken et al. demonstrated the electrodeposition of columnar lithium in a LiTFSI‐based electrolyte using trace amounts of water as an additive (50 mM).^[^ ^164^ ^]^ Notably, it is shown that 25 mM of trace water and lithium salt under a specific ratio in the LiTFSI‐based electrolyte is beneficial in obtaining uniform dense Li deposits, forming an effective LiOH/Li_2_O passivation layer. Using LiClO_4_‐based electrolyte also works as it helps to form an analogous LiOH/Li_2_O passivation layer on the surface.

An areal capacity of 1 mAh cm^−2^ corresponds to ≈5 µm of Li metal, assuming that it is fully dense. However, the Li deposition process is sensitive to electrolyte components, the substrate, and the electrochemical procedure, making it challenging to fabricate a dense and thin Li layer. In addition to electrolyte modifications, modifying separators and Cu substrates to achieve homogeneous Li⁺ deposition represents a feasible approach. For instance, Li et al. coated MnCO_3_ on a polypropylene separator (PP/MnCO_3_) to sustain the uniform Li nucleation effect.^[^ ^170^ ^]^ The in situ formed Mn nanoparticles on the anode helped reduce the nucleation overpotential and achieve dendrite‐free, large‐grain‐size nodular Li deposition at a current density of 1 mA cm^−2^. The 10‐µm‐thick sheet‐like Li showed excellent cycling stability, with a capacity retention of 97.4% over 250 cycles in an LFP full cell (N/P ≈ 3.5) using a gel electrolyte. Considering the rigorous demands on electrodeposition devices for Li metal, co‐deposition of lithium alloys is rarely reported. In situ alloying with electrodeposited Li and substrates is a feasible approach. Yang et al. reported using a Zn layer electrodeposited on a Cu disk as a precursor, with subsequent Li deposition to form the Li–Zn alloy.^[^ ^171^ ^]^ Recently, Kim et al. reported that a Cu substrate coated with Ag nanoparticles could form a uniform 20‐µm‐thick Li‐Ag alloy via an in situ alloying reaction during Li deposition (Figure 9g).^[^ ^165^ ^]^ A LiNO_3_‐containing electrolyte enabled the formation of a robust SEI comprising LiF and Li_2_O with high mechanical strength, and Li_3_N with high ionic conductivity. Preloading Li onto the modified substrate through such electrolytes produces a 20‐µm‐thick Li anode. Consequently, the ASSBs assembled with the Li−Ag‐alloy‐loaded Cu foil, paired with Li_6_PS_5_Cl SSE and an NCM cathode, exhibited good cycling performance over 300 cycles at room temperature. Those results are illustrated in Figure 9h. **Table** **3** summarizes the electrochemical performances of LMAs that were fabricated by various solution‐based wet methods.

**Table 3:** Summary of electrochemical performances of thin Li metal anodes fabricated by various solution‐based wet methods.

Materials type	Processing method	Electrochemical performances			Refs.
		Thickness (area capacity)	Half‐cell performances (electrolytes)	Full cell performance (cathode, N/P ratio, size)	
LMP	Slurry coating Li powder with LiNO_3_ additive on Cu	20 µm (≈4 mAh cm^−2^)	3 mA cm^−2^,1 mAh cm^−2^, 200 h (LiFSI in DME‐TTE 1:1.2:3)	Coin cells 81.8%@200 cycles, C/3 (1.82 mAh cm^−2^ NCM811, **N/P = 1.9**)	[154]
LMP	Slurry coating of Li powder on Cu@C substrate	20–40 µm (≈4–8 mAh cm^−2^)	1 mA cm^−2^,1 mAh cm^−2^, 200 h (1 M LiTFSI in DME/ DOL 1:1 vol + 0.2 M LiNO_3_)	Coin cells 96%@250 cycles, 0.5C (1.82 mAh cm^−2^, LMO, **N/P = 8.8, N/A**)	[159]
LMP	Slurry coating Li powder with AgNO_3_ additive on Cu	40 µm (8 mAh cm^−2^)	1 mA cm^−2^,1 mAh cm^−2^, 200 h 1.15 M LiPF_6_ in EC/EMC (3:7)+2wt.% FEC	Coin cells 85.8%@500 cycles, 1 C‐ 3 C (1.35 mAh cm^−2^, NCM622, **N/P = 6, NA**)	[172]
Li_22_Si_5_	Li_22_Si_5_‐based slurry coating on Cu	5 µm (0.34 mAh cm^−2^)	2 mA cm^−2^, 1 mAh cm^−2^, 600 h (LiFSI‐DME‐TTE 1:1.2:3 mol)	Single‐layer pouch cell 90%@100cycles, 0.5C (4 mAh cm^−2^ NCM811, **N/P = 0.085,** ≈**5 × 6 cm^2^**)	[160]
ED‐Li	Electro‐deposition of Li onto Cu	10 µm (≈2 mAh cm^−2^)	3 mA cm^−2^, 1 mAh cm^−2^, 1300 h (1 M LiTFSI in DME/ DOL 1:1 vol + 1% LiNO_3_)	Coin cells ≈50%@300 cycles, 0.2C (≈1.7 mAh cm^−2^, **N/P = 1.2, N/A**)	[173]
ED‐Li	Electro‐deposition of Li onto Cu	20 µm (4.13 mAh cm^−2^)	5 mA cm^−2^, 1 mAh cm^−2^, 140 h (LiFSI‐DME‐TTE 1:1.2:3 mol)	Coin cells 80.0%@200 cycles, C/3 (3.8 mAh cm^−2^ NCM622, **N/P =** 1.1)	[162]
ED‐Li	Electro‐deposition of Li onto Cu	10–20 µm (≈2–4 mAh cm^−2^)	5 mA cm^−2^,1 mAh cm^−2^, 2000 h (1 M LiTFSI in DME/ DOL 1:1 vol + 1% LiNO_3_)	Coin cells 97.4%@250 cycles, 0.75 C (0.65 mAh cm^−2^, LFP, **N/P =** 3.1**, N/A**)	[170]
ED‐LiAg	Electro‐deposition of Li and Ag onto Cu	20 µm (≈4 mAh cm^−2^)	0.6 mA cm^−2^,0.6 mAh cm^−2^, 2000 h (Li_6_PS_5_Cl solid‐state‐electrolyte)	Coin cells ≈80%@300 cycles, 0.3C (2.4 mAh cm^−2^, NCM, **N/P =** 1.6**, N/A**)	[165]

In summary, slurry coating of powder‐based Li represents a feasible means to fabricate ultrathin Li‐electrodes compatible with current LIBs setups. However, a critical hurdle lies in the high sensitivity of powdered Li metal to moisture and air, imposing high costs in handling, storing, and transportation. Unlike bulk compact Li foil, the galvanic corrosion between Cu and Li may be more severe with Li powder‐based electrodes. Electrodeposition of metallic Li allows precise control over thickness down to a few microns and offers improved Li purity and compactness. High temperatures are largely avoided. However, unlike conventional wet‐processing, processing of metallic Li‐films requires a dry‐room atmosphere and potentially hazardous organic electrolytes, making it less likely to be commercially viable. Due to the large difference in the electrochemical reduction potential of Li and everything else (apart from Na and K), there are few reports of co‐electrodeposition of Li alloys. Rather, post‐deposition alloying appears more feasible, e.g., electrodepositing Li on an alloying support and then annealing the bilayer. The electrodeposit quality, i.e., uniformity, morphology, and surface chemistry, is heavily affected by the electrolyte formulation, substrate properties, and the plating current. Low current density is often required to obtain dense Li layers, hence the low production efficiency. Moreover, a low electrodeposition rate could lead to more electrolyte impurities being entrapped or reacted within the Li electrodeposit, e.g., from water molecules entrapped in a non‐aqueous solvent. Even at the lab scale, the electrochemical pre‐lithiation process is complex and time‐consuming for sample preparation and battery evaluation. At an industrial scale, fire safety and environmental contamination issues may also exist when employing a large bath of non‐aqueous solvent.

## 5 Physical Vapor Deposition (PVD)

### 5.1 Basics of Thin Film Growth

Before discussing specific studies concerning physical vapor deposited (PVD) Li thin films, it is useful to consider the basics of the early stages of thin film growth. While the related theory was largely developed in relation to adatoms depositing on a substrate from a vapor phase, the general concepts likewise pertain to the early stages of electrodeposition and even solidification. Effectively, if there is an underlying support, and especially if this support is crystalline with a *fcc* or *bcc* structure, there will be well‐defined orientation relations between it and the crystallites of Li metal.

Body‐entered cubic polycrystalline thin films and metallic sheets, such as Li foils, normally possess a fiber texture.^[^ ^174^, ^175^ ^]^ This is when one (110), (100), or (112) plane of the crystallite is aligned parallel to the substrate surface while being rotated around the substrate surface normally. For example, for magnetron‐sputtered Cr films, the initial texture has been reported to be primarily (110) or (100).^[^ ^176^, ^177^ ^]^ Since the rolled fcc Cu current collector foils are also polycrystalline, a series of well‐defined orientation relationships (ORs) may be possible between the individual Li grains and the individual Cu grains. Several bcc (Li) – fcc (Cu, Ni) ORs are well‐known, having been studied extensively concerning phase transformations in steels, nickel alloys, and Fe‐Cu alloys.^[^ ^178^, ^179^, ^180^, ^181^ ^]^ The most well‐known ORs are the Kurdjumov–Sachs K–S (110)bcc//(111)fcc and [111]bcc//[110]fcc, and the Nishiyama–Wasserman N–W (110)bcc//(111)fcc and [100]bcc//[110]fcc. There is also the Invariant Line IL OR, which predicts a common direction corresponding to a vector of least distortion that is between [111]bcc and [110]fcc while maintaining the (110)bcc//(111)fcc plane matching.^[^ ^92^ ^]^ All the ORs are frequently reported for solid‐state precipitation reactions, and are generally based on plane (K‐S, NW) or directional (IL) matching.

In the case of the deposited Li metal–support ORs, both chemical energy and strain energy considerations are important. In general, there is no direct epitaxial relationship between the Li and its support. It should also be noted that modern density functional theory (DFT) simulations that consider the chemical interface energy of Li─Cu result in similar ORs, with an emphasis on (110)bcc//(111)fcc plane matching. A collective fiber texture of the film and of the support is consistent with the presence of well‐defined ORs between the individual crystals of bcc Li metal and fcc Cu. It indicates that collectively, there is a preferred orientation based on plane matching.

After the initial layer of crystalline Li is deposited by PVD onto the metal or carbon support, the next layer of Li grains (crystallites) will interact exclusively with existing Li, as the support is now covered. This assumes complete wetting and no exposed underlying Cu or other support materials. Lithium nucleation and growth will subsequently occur on pre‐existing Li metal, with the bcc//fcc OR considerations no longer applicable. During deposition, there will be additional Li grain on Li grain nucleation, as well as grain growth throughout the entire film. If there are significant stresses involved during growth, likely, the dynamic recrystallization will also occur, potentially altering the film texture as a result.

During deposition, the growth velocity of the Li grains will depend on their crystallographic orientation relative to the incoming atomic flux from magnetron sputtering or thermal evaporation. It has been reported that the activation energy for Li adatom diffusion on the Li (110) is about half the value it is for Li (100), being 0.046 and 0.09 eV, respectively. This will cause a significantly faster adatom diffusion on the Li (110) terminating grain surfaces vs on other surfaces such as (110) or (112).^[^ ^182^, ^183^ ^]^ Lithium adatoms will diffuse faster on the closer‐packed (110) relative to other grain faces, resulting in those facets growing at a faster rate relative to the incoming atomic flux. As the physical vapor deposited film thickens, the more rapidly growing (preferably oriented) grains will subsume the slower‐growing, less preferably oriented ones. The slower‐growing (100) and (112) terminating grains will become subsumed by the faster‐growing (110) grains. This competitive grain evolution, which should occur with Li film thickening, is known as Van der Drift film growth. It has been confirmed experimentally and through modeling to be a key aspect of PVD polycrystalline metal film microstructure.^[^ ^184^, ^185^, ^186^, ^187^ ^]^ In fact, Van der Drift grain growth is widely reported for deposited polycrystalline metals, semiconductors, and oxide films.^[^ ^188^, ^189^ ^]^

This evolutionary growth mechanism, which occurs during thin film PVD and also during thin film electrodeposition and solidification, causes the film texture to progressively sharpen with film thickening. In parallel, as the film thickens, the average grain size of the Li deposit increases, causing the electrode's terminating surface to become geometrically rougher. The film's surface morphology is expressed as root‐mean‐square roughness and is measured by AFM. For an example of this approach, consider a recent study on grain size–surface roughness relations for a milled and compacted solid‐state electrolyte.^[^ ^190^ ^]^ It is known that the average surface roughness of a polycrystalline film scales approximately with the average grain size in the vicinity of the surface. Therefore, as the Li film thickens and the grain size increases, its surface roughness increases proportionally as well. This may have significant implications for electrochemical stability during cycling, for example, providing a rationale to the common observation that thick (e.g., 500 µm) Li foils will cycle worse than thinner (e.g., 25 µm) ones. Multiscale modeling has conclusively shown that during electrodeposition/dissolution, electrode surface roughness drives local field and stress concentrations, which in turn promote an unstable SEI and dendrite growth.^[^ ^191^, ^192^, ^193^ ^]^

For vapor‐deposited materials (and also for electrodeposits), the sharpening of texture is known to be accelerated with increasing deposition rates. Meanwhile, an increased PVD deposition rate (and also lower temperature) promotes a smaller average grain size and corresponding surface roughness.^[^ ^111^ ^]^ Increasing deposition rate (PVD, solidification, electrodeposition) promotes more copious Li grain nucleation due to a higher driving force and less time for diffusion to occur.^[^ ^112^ ^]^ Studies on Li metal electrodeposition have shown that film‐preferred orientation is directly related to electrochemical stability.^[^ ^113^ ^]^ The (110) texture or preferred orientation is well correlated with improved electrochemical stability during extended cycling. Therefore, there strong relationship between processing (deposition rate, film thickness), microstructure (crystallographic texture, grain size, surface roughness), and electrochemical stability of Li anodes during extended cycling. In our opinion, a more in‐depth understanding of these links is needed for the metal anode field to advance. This is especially the case regarding the PVD deposition process, where the effects of processing on the anode microstructure and stability have not been extensively analysed.

### 5.2 Thermal Evaporation

**Figure 10:**
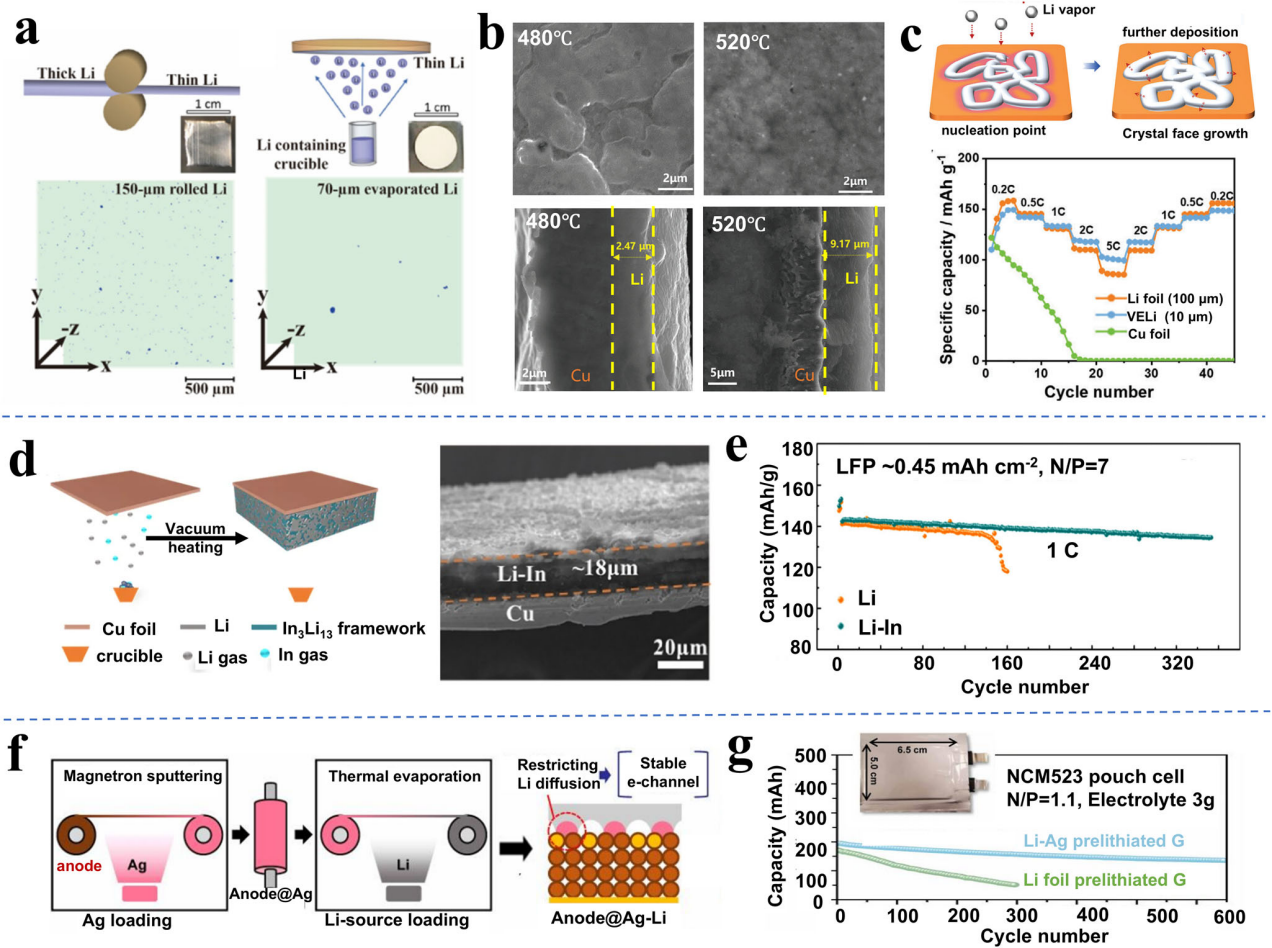
Vacuum engineering for Li vapor deposition. a) X‐ray microtomography images comparing the surface contaminant concentrations on rolled and thermal evaporated Li foils.^[^ ^196^ ^]^ b) SEM images of evaporated Li layer with thickness tuned from 2 to ≈10 µm through control of the evaporation temperature. c) Thermal evaporation leads to out‐of‐plane structures of the Li‐layer, giving improved cycling and rate performance in LMBs.^[^ ^197^ ^]^ d) Schematic and SEM images of a thin Li‐alloy layer by vacuum co‐evaporation of Li and In. e) Full cell cycle performance of a Li‐In alloy vs neat Li.^[^ ^198^ ^]^ f) Illustration of pre‐sputtering a layer of Ag to promote uniform evaporation of Li onto graphite, yielding a thin Li−Ag alloy (50 at.% Li) layer for pre‐lithiation, g) Cycle life of NCM||graphite pouch cells adopting Li‐Ag or neat Li as pre‐lithiation sources. Panel a reproduced with permission: Copyright 2022, American Chemical Society. Panels b and c reproduced with permission: Copyright 2024, Wiley. Panels d and e reproduced with permission: Copyright 2020, Elsevier. Panels f and g with permission: Copyright 2024, Wiley.^[^ ^201^ ^]^

Physical vapor deposition (PVD) has gained attention due to its capability of achieving thin Li layers with precise control of thickness down to the nanometer scale.^[^ ^114^ ^]^ Lithium film impurities will then influence electrochemical behavior in ways that are difficult to predict ahead of time. Thin (typically sub‐5 µm) films of highly pure Li can be deposited by thermal evaporation due to its low melting point (T = 180.5 °C) and high vapor pressure (p = 10^−4^ Torr at T = 407 °C). Compared to ingot‐based rolling, the vapor‐based deposition of thin Li foil offers superior surface quality.^[^ ^194^ ^]^ Earlier studies by Yamakawa et al. reported a vapor deposition method to produce Li thin films on 9 cm^2^ Cu and PP substrates, with thicknesses ranging from 1 to 20 µm.^[^ ^195^ ^]^ X‐ray tomography examination from Westover's group identified that rolled Li metal films had an impurity concentration of ≈1300 particles mm^−3^, while thermal evaporated Li‐metal films had an average impurity concentration of ≈19 particles mm^−3^.^[^ ^196^ ^]^ Those results are illustrated in **Figure** **10**a. Zhang's group demonstrated that the thickness of the Li metal layer can be precisely controlled by adjusting the evaporation temperature.^[^ ^197^ ^]^ Ultrathin Li foils with thicknesses ranging from 2 to 10 µm were successfully prepared, exhibiting high purity and adequate adhesion to the Cu substrate. Deposited at 480 °C, the deposited Li films exhibit a relatively smooth surface morphology, as illustrated in Figure 10b. Deposited at 520 °C, the film appears rougher due to de‐wetting from the support. As shown in Figure 10c, these spontaneously formed patterns increase the active surface area of Li‐films, delivering improved rate performance as compared to rolled Li, and achieving fast charge rates (up to 5C).

Alloy films can also be fabricated by co‐evaporating or by co‐sputtering from different sources. While it is possible to obtain a relatively accurate alloy film composition by sputtering from a single alloy target, co‐evaporating from a single crucible is not advised due to the dissimilar melting points and vapor pressures of the elements. Gao et al. reported a thin Li/In_3_Li_13_ anode with a thickness of 18 µm, prepared through vacuum co‐evaporation of Li and In.^[^ ^198^ ^]^ Those results are illustrated in Figure 10d. The Li─In alloy films were shown to improve the cycle life of LFP full cells to over 300 cycles, as opposed to pure Li counterparts that failed after 160 cycles (Figure 10e). Another group manufactured a novel Li/Li_x_Sn thin film by sequential deposition of magnetron sputtered Sn (≈200 nm) and thermally evaporated Li (500–1000 nm).^[^ ^199^ ^]^ Zhang and Yan et al. compared Li foils (1.0–4.0 µm), prepared by mechanical rolling or by vacuum evaporation onto graphite anodes, as pre‐lithiation sources.^[^ ^200^ ^]^ The evaporated Li foil displayed more conformal contact with the graphite interface, facilitating the pre‐lithiation kinetics. The evaporation‐based pre‐lithiation appeared to be much faster and showed high Li utilization up to 91.0%. In contrast, with rolling‐based pre‐lithiation, a large amount of dead Li accumulated at the anode interface and leading to increased polarization.

Authors found that ≈4 µm Li─Ag alloy foil (50 at. % Li) was even more ideal as a pre‐lithiation source, adopting sequential magnetron sputtering of Ag and thermal evaporation of Li, per Figure 10f.^[^ ^201^ ^]^ The Li−Ag alloy interlayers, when coated onto the graphite anode, offer superior active‐Li utilization (90.7% vs 61.2% of neat Li) due to the improved electron channels. Per Figure 10g, this results in a favorable NCM523‐graphite pouch cell cycle life, with capacity retention of 95.8% over 600 cycles. Winter et al. sputter‐coated Li metal electrodes with lithiophilic gold (Au) or zinc (Zn).^[^ ^202^ ^]^ It is shown that the presence of the corresponding Li‐intermetallic phase (Li_15_Au_4_, LiZn) at the surface of the Li metal electrodes results in a more homogeneous dispersion of the Li deposits, and improves overall cycling stability with lower overpotentials. This highlights the sputter coating of the thin layer of lithiophilic Li‐alloys as a promising approach to improve the performance of lithium metal anodes.

### 5.3 Magnetron Sputtering and Pulsed Laser Deposition

**Figure 11:**
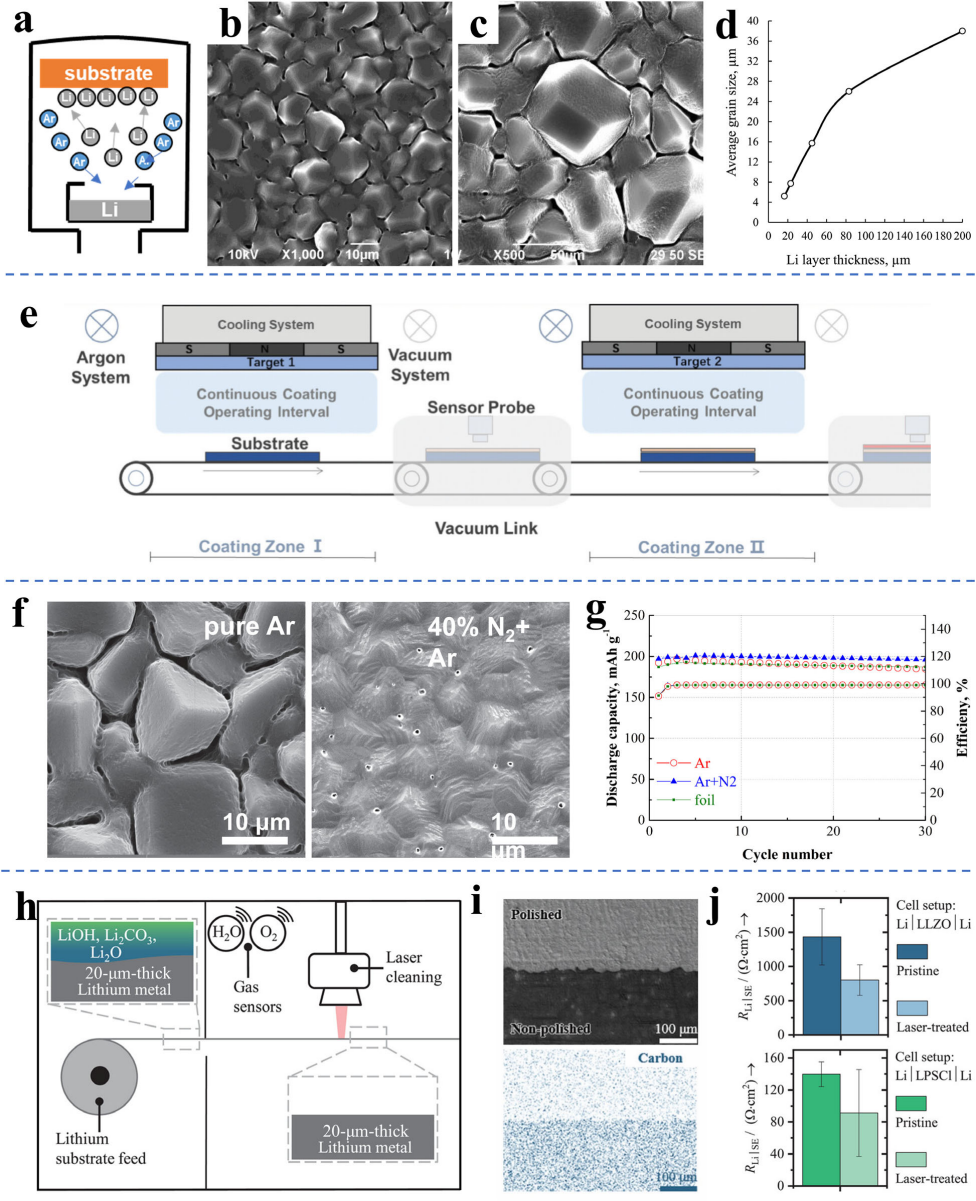
a) Schematic of magnetron sputtering to deposit Li, (b,c) SEM images of the sputtered 40 µm b) and 200 µm c) Li‐layer,  d) The thickness of sputtered Li is proportional to grain size.^[^ ^203^ ^]^ e) Schematic depicting the continuous sputtering for large‐scale electrode fabrication.^[^ ^204^ ^]^ f) SEM images of sputtered Li using Ar (left) and N_2_+Ar mixture (right). g) Full cell performance adopting Li sputtered using Ar or Ar+N_2_, and conventional Li foil.^[^ ^205^ ^]^ h) Schematics of applying a picosecond laser to polish the 20 µm rolled Li‐foil. i) SEM image of laser‐treated Li surface, j) Li|SSE interfacial resistance employing pristine and laser‐treated Li. Panels a to d reproduced with permission: Copyright 2019, Elsevier. Panel e reproduced with permission: Copyright 2024, Wiley. Panels f and g reproduced with permission: Copyright 2023, The Electrochemical Society. Panels h to j reproduced with permission: Copyright 2024, Wiley (open access).^[^ ^206^ ^]^

Lithium metal may be deposited by both magnetron sputtering and thermal evaporation, both techniques having widespread utilization in numerous industries. Magnetron sputtering utilizes the gas discharge phenomenon that occurs when a voltage is applied between the target (cathode) and the substrate (anode) under a vacuum of 10^−2^–10^1^ Pa. When charged ions (often Ar^+^) are bombarding the target materials, atoms detach and escape from the target surface. Driven by the toroidal magnetic field that is perpendicular to the substrate, these escaped atoms form deposits at the substrate surface that oppose the target. The deposition process is line‐of‐sight, which makes sputtering well‐suited for planar substrates but not ideal for complex 3D geometries, including when substrate porosity is present. An overview of the magnetron sputtering process is shown in **Figure** **11**a. Kolosnitsyn et al. employed a lithium liquid‐phase target to facilitate the Li‐deposition in magnetron sputtering.^[^ ^203^ ^]^ As shown in Figure 11b–d, the grain size of the Li films is proportional to the sputtering time, agreeing with the earlier discussed point that grain size scales approximately with the film thickness.^[^ ^207^ ^]^ Implemented into symmetric cells, the resistance of magnetron‐sputtered Li film is much lower than that of conventional rolled Li foil. This may be due to some reasons, including a thinner native passivation layer. A lower Fe impurity content could also be responsible for this effect, since Fe (derived from the rollers) is known to catalyse SEI growth.

Liu et al. proposed an equipment design for a continuous sputtering process that can enable large‐scale electrode fabrication.^[^ ^204^ ^]^ A schematic illustrating this process is shown in Figure 11e. A key element involved in such a technological process is the large‐space vacuum system that allows sputtering operation in a semi‐continuous manner, with intermittent sputtering and continuous conveyor flow. Multilayered deposition may be achieved by the transfer of samples through a series of vacuum links. Another advantage of sputtering is the possibility of tuning Li film morphology/chemistry by introducing reactive gases into the plasma. Kolosnitsyn et al. used a mixture of N_2_ and Ar in the plasma and achieved more uniform Li deposition.^[^ ^205^ ^]^ The grain sizes of lithium decrease with increased N_2_ content, with the argument that the formed Li_3_N pins grain boundaries. This is illustrated in Figure 11f. When tested in full cells with an NCM cathode, a 35 µm thick Li film produced by sputtering displayed improved capacity retention vs a 100 µm thick commercial Li foil (Figure 11g).

Pulsed Laser Deposition (PLD) is another PVD method for preparing thin films using high‐energy laser pulses. Pulsed laser deposition (PLD) has also been employed for the fabrication of battery materials.^[^ ^208^, ^209^ ^]^ Currently, PLD is used for fabricating micro‐batteries, where each battery component (cathode, solid electrolyte, and anode) is deposited in a layer‐by‐layer sequence.^[^ ^210^, ^211^ ^]^ For example, Tarascon's group employed a KrF excimer laser to deposit thin lithium layers onto 2 cm^2^ stainless steel supports at room temperature. While in principle the PVD films should be extremely pure, in practice, impurities are also present.^[^ ^212^ ^]^ In earlier research, thin Li films ranging from 500 nm to 3 µm were prepared by adjusting PLD time, achieving a range of morphologies that depended on the film thickness (rougher for thicker films).^[^ ^212^ ^]^ While PLD is not ideal for manufacturing due to its high cost and limited processing area, laser processing can be employed for surface polishing of Li foils fabricated by other techniques. Kriegler et al. developed a picosecond‐pulsed laser to modify the surface of lithium metal foils.^[^ ^206^ ^]^ Laser surface treatment led to a reduction of oxygen and carbon by ≈80% (likely due to the removal of residual oils and Li_2_CO_3_), as visualized by EDX mappings shown in Figure 11h. Of course, EDX analysis is a qualitative tool, especially when it comes to analyzing carbon and oxygen content near a metal surface. However, the overall trend is believable, with laser treatment being effective in removing the metal's top surface layer, which is the most contaminated. The interfacial contact between the Li foil and the solid electrolytes, including both garnet oxide LLZO and sulfide Li_6_PS_5_Cl, is improved after such surface treatment. Those results are illustrated in Figure 11i,j.

In our opinion, one of the more interesting questions to be explored concerns the role of the support “memory effect” in the microstructure/morphology of the deposited films. After the first monolayer of Li crystallites forms on a given support, the next layer will only “see” this metal surface, rather than the underlying support. A memory effect is when the initial wetting behavior of the first few layers of PVD Li metal on the support continuously influences the morphology and texture of the thickening deposit. A related question concerns how this initial microstructure then affects the electrochemical properties of the anode. A substrate memory effect is routinely reported in the literature, that the substrate properties influence both the microstructure of the fabricated lithium film and its electrochemical performance, even after many cycles.^[^ ^213^, ^214^ ^]^ One can even argue that such an effect is taken for granted; researchers assume that it will be the case, despite a satisfactory explanation for its origin. A comparison of the various PVD prepared Li anodes is provided in **Table** **4**.

**Table 4:** Summary of thin Li metal anodes that were fabricated by PVD methods.

Materials type	Processing method	Electrochemical performances			Refs.
		Thickness (area capacity)	Half‐cell performances (electrolytes)	Full cell performance (cathode, N/P ratio, size)	
VE‐Li	Vacuum evaporation of Li onto Cu	9.17 µm (1.82 mAh cm^−2^)	0.5 mA cm^−2^,0.5 mAh cm^−2^, 200 h (1 M LiPF_6_ in EC: DMC 1:1 vol + 5% FEC)	Single‐layer pouch cells ≈100%@50 cycles, 1C (≈1 mAh cm^−2^ LFP, **N/P = 1.9, 6 × 11 cm^2^**)	[197]
Li@Li_2_CO_3_	Vacuum evaporation in Ar/CO_2_ mixture atmosphere	30–70 µm (≈4–8 mAh cm^−2^)	0.025 mA cm^−2^,0.5 mAh cm^−2^, 900 h (LiTFSI/PEO solid‐state‐electrolyte)	N/A	[196]
Li/Li_x_Sn_y_	Magnetron sputtering of Sn and vacuum evaporation of Li on Cu	1 µm (≈0.2 mAh cm^−2^)	0.025 mA cm^−2^,0.025 mAh cm^−2^, 350 h (1 M LiPF_6_ in EC/DEC/EMC 1:1:1 vol)	N/A	[199]
Li‐In	Vacuum co‐evaporation of Li and In onto Cu	18 µm (3.81 mAh cm^−2^)	0.5 mA cm^−2^, 0.5 mAh cm^−2^, 230 h (1 M LiPF_6_ in EC/DMC 1:1 vol)	Coin cells ≈96%@350 cycles, 1C (≈0.5 mAh cm^−2^ LFP, **N/P =** 7**, N/A**)	[198]
Li	PLD polished Li	20 µm (≈4 mAh cm^−2^)	0.1 mA cm^−2^, 0.1 mAh cm^−2^, 70 h (LPSCI solid‐state‐electrolyte)	N/A	[206]
MS‐Li	Magnetron Sputtering onto Cu	10–76 µm (2–15 mAh cm^−2^)	(1 M LiPF_6_ in EC/PC 1:1 vol)	Coin cells ≈100%@30 cycles, 0.2C (≈1 mAh cm^−2^ NCM523, **N/P =** 2**, N/A**)	[205]

For PVD Li metal films, why (and how) do the support‐deposit energetics matter beyond the first layer? This is also relevant for subsequent electrochemical cycling: How is electrodeposition/dissolution behavior influenced by support‐electrodeposit energetics even when not cycled at 100% depth‐of‐discharge (thereby exposing the support)? One can hypothesize that what is in play is the role of metal epitaxy and the inherited grain size. After the initial Li layer is deposited, the subsequent metal should grow epitaxially with growth velocities depending on the crystal orientation, while forming additional nuclei as the film thickens. This type of Van der Drift film was discussed previously in the article. Whether this hypothesis is true, and if so, the phenomenology of the Van der Drift film growth process, is to be determined. From a practical battery application perspective, this question is paramount: For the vast majority of applications, any secondary battery is not run to full discharge. For example, EVs don't operate to full battery discharge, which is equivalent to running gas powered vehicle to full empty. Therefore, why do the support energetics (during solidification, PVD, electrodeposition, etc.) have such an outsized influence on the electrochemical stability of anodes that are not fully electrodissolved at each cycle?

## 6 Advanced Microstructural Analysis

### 6.1 Cryogenic Bulk and Surface Analysis

Characterizing Li microstructures in their pristine and original state remains a major challenge due to the extremely sensitive nature of Li metal and its native SEI to both electron beams and atmospheric exposure. To address this, cryogenic techniques have been developed to suppress reactivity and enhance beam stability, allowing the analysis of morphological and crystallographic properties using cryo‐FIB‐SEM, cryo‐TEM, and cryo‐XPS. These methods have significantly advanced our understanding of Li and its SEI. However, the cryogenic vitrification process itself may introduce artifacts such as phase transitions, microstructural distortion, or sample degradation, issues that remain underexplored within the community. Furthermore, access to cryo‐based characterization tools is often limited, and logistical delays of days to even months before analysis can allow undesirable aging of samples. Such temporal gaps may result in subtle structural changes that are difficult to discern or disentangle from natural storage effects. Sample transportation further exacerbates these issues, as even minor temperature fluctuations or improper sealing can lead to unintentional exposure to air and moisture, compromising sample fidelity.

**Figure 12:**
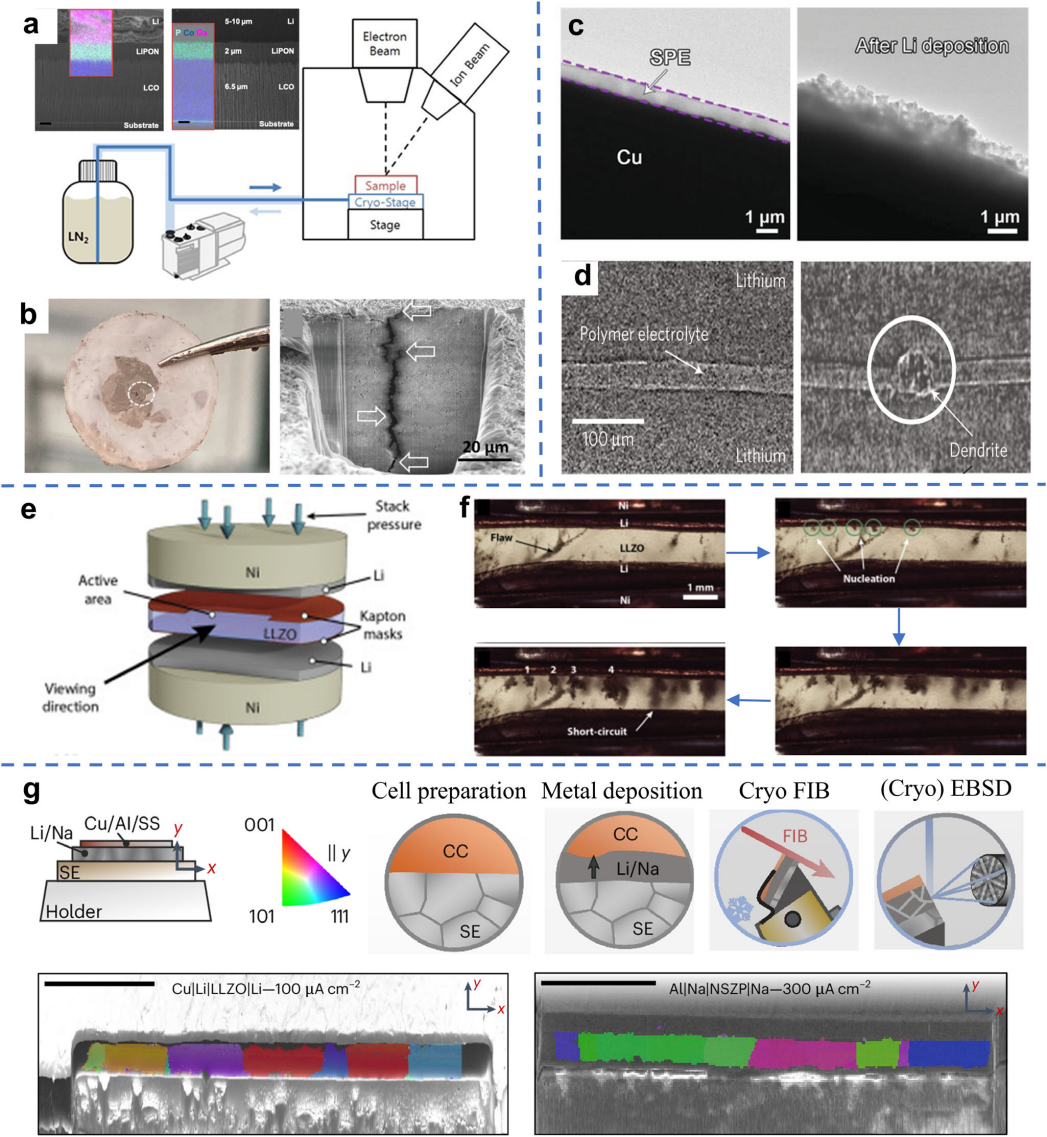
a) Schematic of cryogenic focused ion beam (cryo‐FIB) system, and the cross‐section SEM images and EDS analysis of Li|LiPON|LCO obtained by FIB milling at room temperature(left) and cryogenic temperature(right).^[^ ^215^ ^]^ b) Photograph of the PP separator with a 1.5 mm center hole, and the cryo‐FIB‐SEM images of the corresponding area capture the path of the dendrite penetrating the solid‐state argyrodite electrolyte. c) Cryo‐TEM characterization of Li/PEO interface (left) and after Li deposition (right).^[^ ^216^ ^]^ d) X‐ray tomography slices showing the cross‐sections of pristine (left) and cycled (right) symmetric Li cells using polystyrene‐block‐poly(ethylene oxide) copolymer electrolyte (SEO).^[^ ^217^ ^]^ e) Schematic of operando cross‐sectional LLZO‐based solid battery cells. f) Operando optical microscopy capturing the dynamic growth of dendritic Li penetrating the SSE.^[^ ^218^ ^]^ g) Recent demonstration of combined cryo‐FIB and EBSD analysis on the microstructures of the electrodeposited Li and Na metal. Panel a reproduced with permission: Copyright 2019, American Chemical Society. Panels b and c, reproduced with permission: Copyright 2020, Wiley. Panel d, reproduced with permission: Copyright 2014, Springer Nature. Panels e and f, reproduced with permission: Copyright 2020, Cell Press. Panel g reproduced with permission: Copyright 2024, Springer Nature.^[^ ^219^ ^]^

The significant morphology and SEI content change that Li electrodeposits undergo during cycling present substantial challenges for characterization. Post‐cycled specimens are much harder to characterize with minimal artifacts as compared to as‐fabricated ones. Thin Li foils exhibit high chemical reactivity, which makes them highly susceptible to contamination by the external atmosphere during sample transfer and by residual gas in the chamber. Moreover, due to its low density and melting point, Li is susceptible to electron beam damage, especially at high accelerating voltages such as 200 or 300 KV, typical of TEM analysis. Cryogenic electron microscopy (cryo‐EM) and cryogenic focused ion beam (cryo‐FIB) milling techniques have gained widespread attention in the battery materials field since they allow analysis in a less damaging environment than at ambient temperature. Specialized holders allow for liquid nitrogen cooling of the sample, maintaining it at ≈−170 °C during electron beam analysis or Ga^+^‐ion sectioning. In a seminal work, Meng et al. utilized FIB at room temperature and cryogenic temperature to investigate the metal and the SEI structure of cycled Li anode from a Li|LiPON|LCO cell.^[^ ^215^ ^]^ As shown in **Figure** **12**a, the cryo‐FIB method reduced the degree of beam damage, allowing analysis of microstructural features that would be otherwise obscured. Each component and interface in the cell can be imaged, highlighting the thin 5–10 µm Li layer and 2 µm LiPON.

Mitlin's group designed a cell involving a polypropylene (PP) separator featuring a 1.5‐mm‐diameter hole between two SSE layers.^[^ ^190^ ^]^ Figure 12b (left) provides an optical photograph of the PP separator that was specially designed to capture the location where the dendrite has penetrated in the SSE in the Li metal‐argyrodite half‐cell. As shown in Figure 12b (right), the Li metal dendrite is a 2D “curtain‐like” sheet, rather than a 1D filament, that snakes between the Li_6_PS_5_Cl grains and fills the interparticle pores. Moreover, the dendrite is branched, with multiple paths across the electrolyte compact. Around its edges, the Li metal dendrite is reacted with the Li_6_PS_5_Cl, likely forming a combination of Li_2_S, Li_3_P, and Li_3_PS_4_ phases.^[^ ^220^ ^]^ Cryo‐TEM allowed the observation of Li morphology at the onset of nucleation, as shown in Figure 12c.^[^ ^216^ ^]^ A half‐cell consisting of Li metal, a polymer electrolyte (SPE) film, and a Cu grid was employed for the in situ electrodeposition test. Lithium metal grains emerged between the SPE and Cu grid, pushing up the flexible SPE film at the corresponding spot. The irregular Li grain was 1.5 µm in diameter and appeared to be isolated rather than part of a more continuous structure.

### 6.2 In Situ and Operando Methods

In contrast, In situ/operando optical and spectroscopic techniques, such as Raman spectroscopy, optical microscopy, or synchrotron‐based X‐ray methods, allow real‐time monitoring of Li microstructures under ambient and/or operational conditions. Nevertheless, they often require the preparation of ultrathin samples or the use of specialized cell architectures to ensure adequate light or X‐ray penetration. This constraint introduces sample‐to‐sample variation and considerable deviation from practical cells, particularly given the inherent differences in LMA dimensions in these cases. Moreover, the lower stack pressure typically found in customized home‐made Swagelok cells can greatly influence Li microstructures and LMA‐electrolyte interactions, especially when solid‐state electrolytes are employed. The in situ/operando monitoring of pressure‐induced electrochemical reactions was difficult and rarely reported to date.

A half‐cell consisting of Li metal, a polymer electrolyte (SPE) film, and a Cu grid was employed for the in situ X‐ray test. Lithium metal grains emerged between the SPE and Cu grid, pushing up the flexible SPE film at the corresponding area where the Cu grid allows penetration of the probe (X‐ray). For solid‐state batteries where the extraction of cross‐sectional information is both meaningful and feasible, X‐ray computed tomography (XCT) became highly useful. Balsara et al. used hard X‐ray microtomography (micro‐CT) to investigate the Li|PEO|Li cells.^[^ ^217^ ^]^ After cycling at 0.02 mA cm^−2^, the micro‐CT shows the presence of multiple heterogeneities across the thin 30‐µm‐thick PEO layer, per Figure 12d. As shown in Figure 12e,f, operando light optical cells can also be used to visualize the macroscopic Li dendrite growth through LLZO electrolyte.^[^ ^218^ ^]^ This is (partially) owing to the contrast between Li and LLZO under the visual light range.

### 6.3 Cryogenic Tomography

A recently published seminal work by Janek et al. demonstrated imaging of the microstructure of Li and Na metal in anode‐free solid‐state batteries employing electron backscatter diffraction in a cryo‐FIB. The authors were the first to demonstrate a reproducible protocol for analysis of grain size and partial texture of electrodeposited Li and Na metal in an anode‐free configuration, demonstrating a larger‐than‐expected typical film grain size (>100 µm) as well as preferred orientation. Those results are illustrated in Figure 12g.^[^ ^219^ ^]^ Looking forward, advancing the characterization of Li microstructures requires real‐time, multi‐dimensional, and automated analytical platforms that preserve sample integrity while enabling in‐depth insights into electrochemical behavior. Operando techniques that can simultaneously control and monitor temperature, pressure, and electrochemical states are particularly promising for elucidating the structure‐performance relationship and electro‐chemo‐mechanical coupling in Li metal systems. In parallel, artificial intelligence (AI) and machine learning (ML) are emerging as powerful tools to accelerate research in this area. AI‐assisted image analysis can streamline the interpretation of complex microstructural data, while ML models trained on multimodal datasets can uncover trends linking processing conditions to Li morphology and performance. These data‐driven approaches, when integrated with automated experimental platforms, can help optimize design parameters and accelerate the development of scalable, high‐performance thin Li metal anodes.

## 7 Failure Mechanisms

### 7.1 Role of Li Foil Thickness

Dendrite growth is caused by a microstructurally and/or geometrically non‐uniform SEI, and remains one key impediment preventing commercial utilization of LMA's.^[^ ^221^, ^222^ ^]^ Ongoing dendrite growth induces unacceptable impedance rise, electrolyte drying, and possibly internal short‐circuiting.^[^ ^223^, ^224^ ^]^ One view is that LMBs with N/P ratios of 1–2.5 (corresponding to 20–50 µm Li) are optimum for extending cycling life. In cells employing a 100 µm Li foil (N/P ≈ 5), an artificially elevated and stable CE is observed during the initial cycles because the excess lithium compensates for irreversible lithium loss at the cathode.^[^ ^225^ ^]^ However, as cycling proceeds, the lean electrolyte volume can no longer fully wet the highly porous SEI, whose surface area continually increases. This leads to heterogeneous SEI accumulation, loss of ionic pathways, and a progressive rise in cell polarization. The result is increasing heating of the cell and a lower voltage and capacity at discharge.

For example, Nowak et al. employed accelerating rate calorimetry (ARC) and differential scanning calorimetry (DSC) to analyze heat released as a function of Li foil thickness.^[^ ^226^ ^]^ The authors reported that cells with a 100 µm thick Li foil release 2.5 times the total heat of cells with a 20 µm foil, contributing to potential thermal runaway. Therefore, reducing the initial lithium inventory will lower the overall reaction heat. On the other hand, when N/P < 1, active Li may become prematurely depleted due to the relatively low CE of LMBs, especially in solid‐state cells.^[^ ^227^ ^]^ For example, researchers deposited 3 µm Li onto Cu foil by thermal evaporation. When paired with a stable low‐voltage cathode LFP, the cell cycled for 15 cycles, its short lifespan being attributed to Li depletion.^[^ ^228^ ^]^ From a cell energy, cell safety, and cell cost viewpoint, lower N/P ratios should be preferred. However, in practice, the optimal N/P ratio is system‐specific and depends on the particular cell design and electrolyte formulation.

### 7.2 Role of Li Foil Defects: Chemical and Geometrical

When integrating Li foils with existing or designer electrolytes, additional deleterious (and unknown) reactions can result due to the bulk/surface impurities, and due to geometrical defects such as frayed edges. The authors report that certain surface impurity layers (e.g., from the rolling operation) on the Li metal surface will deteriorate the electrochemical performance.^[^ ^229^, ^230^ ^]^ Pre‐existing surface cracks in the Li foil have also been reported to be deleterious for extended cycling, presumably acting as locations for electric field focusing or other localized processes.^[^ ^231^ ^]^ Non‐uniform thickness exacerbates internal pressure imbalances within the cell, particularly in practical pouch configurations, which also leads to reduced cell stability during cycling.^[^ ^232^, ^233^, ^234^ ^]^ The cell's electrochemical performance is also influenced by the process of cutting Li foil into electrode sheets, with edge uniformity being an important parameter.^[^ ^235^ ^]^ Self‐supporting ultrathin Li, due to its sticky and soft nature, is more prone to wrinkling and fracture.^[^ ^236^ ^]^ Therefore, with neat thin self‐standing lithium foil, special handling tools such as PET support film is often required. However, it has also been reported that the overall quality of Li foil can be compromised by surface wrinkling and contamination by the plastic support film (PE/PET).^[^ ^237^, ^238^, ^239^ ^]^

It is a major challenge with LTFs to maintain their surface and bulk purity, as well as their uniform geometry after cutting operations. This translates into potential problems with the electrochemical stability of the anodes during extended cycling or storage. Different process parameters will affect the machinability of Li foil, including temperature, humidity, production power, etc. Commonly, Li‐Cu composite foil has better blanking ability, showing smooth sectioning. A Cu substrate should offer much improved mechanical properties to be cut into varying‐sized electrodes; however, its usability is strongly affected by how well the Li mechanically adheres to Cu during processing. Laser beam‐based cutting under an Ar atmosphere is also implemented, as the conventional cutting die is prone to being contaminated by residual lithium. The process employs a high‐power‐density laser beam to irradiate the electrode sheet. The local high temperature causes the material to melt and ablate, thereby completing the cut. However, it remains uncertain whether lasers can be applied to punch the self‐supporting thin lithium, which is more susceptible to the residual heat of the laser beam, causing the cross‐section to collapse.^[^ ^240^ ^]^

## 8 Summary and Outlook

This review critically examines the current advances of techniques for producing thin lithium metal anodes, a key requirement for realizing practical lithium metal batteries (LMBs). Recognizing that conventional research‐grade foils exceed 250 µm in thickness, which is far thicker than what is needed for real‐world applications, we organize our discussion around four primary fabrication routes: i) ingot extrusion followed by mechanical rolling; ii) metallurgical solidification or casting methods; iii) solution‐based wet processes; and iv) physical vapor deposition (PVD) techniques. For each section, we first provide a foundational overview of the underlying scientific principles. This includes considerations such as the mechanical properties of lithium, interfacial wetting, thermal and chemical compatibilities, and scalability. For instance, during mechanical rolling, lithium's low melting point and extreme malleability result in severe sticking, necessitating the use of lubricants. While these additives reduce surface friction, they often leave behind undesirable organic residues that alter surface chemistry and compromise electrochemical stability. In casting‐based approaches, molten lithium exhibits poor wettability on conventional substrates such as copper. Without a wetting‐promoting interlayer (e.g., silver, zinc, or lithiophilic carbons), the molten metal forms discontinuous, non‐uniform films, which can accelerate dendrite initiation and propagation during cycling.

**Figure 13:**
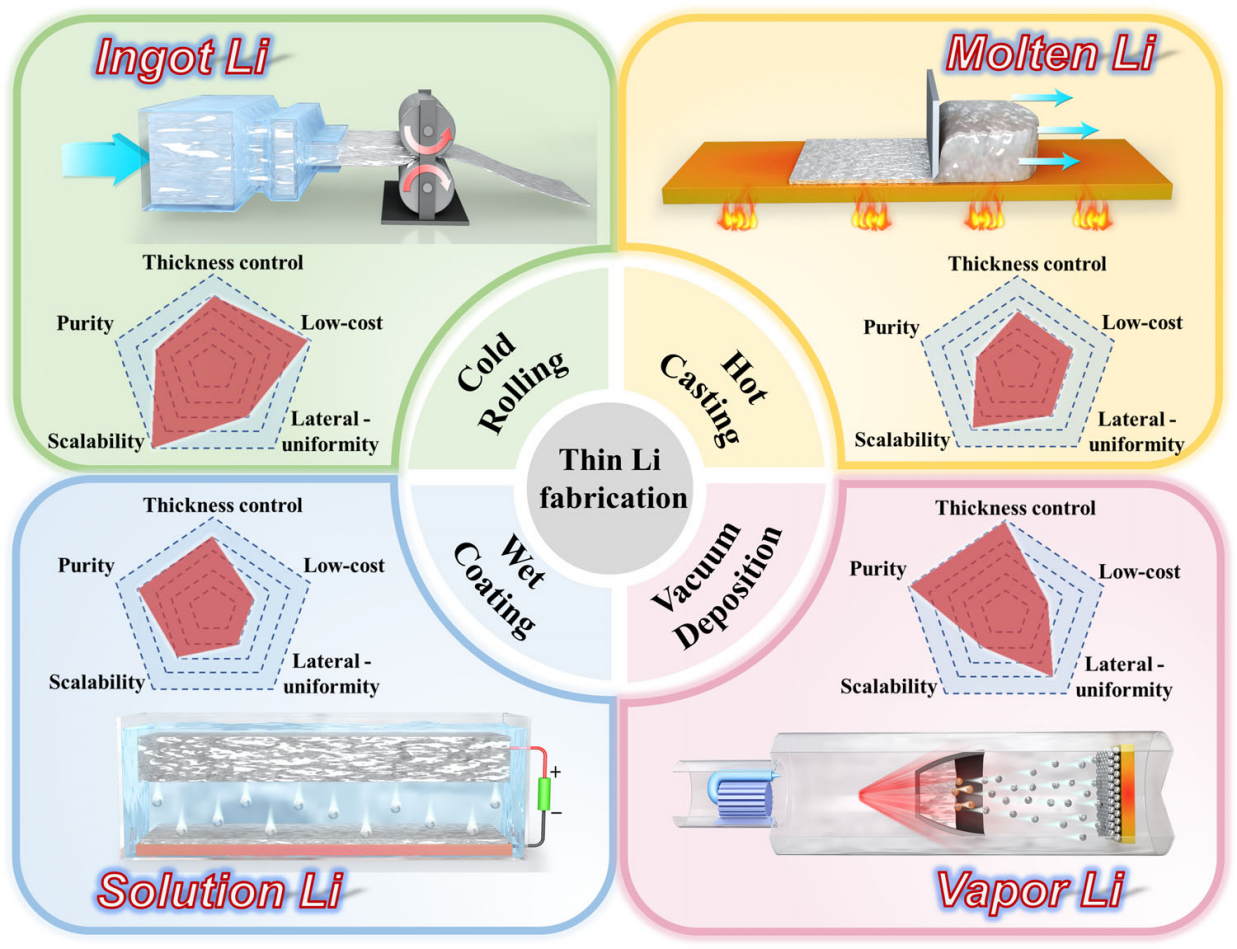
A comparison of the four thin LMA fabrication categories discussed in the review, including a five‐axis spider chart for each one, covering thickness control, low cost, lateral uniformity, scalability, and foil purity.

As a visual summary, we present a comparison of the four LMA processing methodologies, per **Figure** **13**. Extrusion and rolling methods stand out for their straightforward operation and low energy consumption. This translates into a significant cost advantage while delivering superior overall performance across multiple metrics and demonstrating strong scalability for mass production. To date, extrusion and rolling are *the* dominant techniques for commercial‐scale thin Li foil fabrication, with no obvious challenges having yet emerged. With solidification casting methods, maintaining material purity and obtaining a geometrically uniform thin foil are challenging. Molten Li metal is highly reactive, including with water vapor and CO_2_. Moreover, molten Li poorly wets conventional Cu current collectors, since Cu_2_O is lithiophobic. Incorporation of a substrate with tuned Li wettability markedly improves the casting uniformity and holds promise for achieving thinner layers, such as 20 µm and below. Due to the low melting point and high vapor pressure of Li vs nearly all elemental additions, maintaining compositional uniformity of an alloy melt will be extremely challenging at a commercial scale. Precisely regulating the solution coating weight or the amount of electrodeposited lithium ions remains difficult in wet approaches, resulting in inferior product uniformity relative to other methods. Physical vapor deposition excels in uniformity, purity, and thickness control, yet its high cost and stringent experimental conditions create a cost barrier. With magnetron sputtering, uniform alloy films are fully feasible since co‐deposition may be simultaneously performed from multiple sputtering targets. Magnetron sputtering may also be tuned to be a continuous, rather than a batch process, having a history of being used for manufacturing in many industries. In principle, thermal evaporation and PLD of pure Li may also be scaled into a continuous process, although, to the authors’ knowledge, an industrial approach is less mature.

The review provides representative case studies that illustrate innovative approaches to address the challenges of fabricating thin LMAs. These include using alloying elements to modify the lithium's mechanical behavior, creating lithium‐based composites with structural scaffolds, and engineering substrate surfaces with functional coatings to improve film adhesion and uniformity. Across all techniques, a recurring observation is that success in achieving planar, conformal lithium deposition is strongly correlated with improved electrochemical signatures such as Coulombic efficiency, interfacial resistance, and cycling stability. Each section concludes by identifying critical knowledge gaps and open scientific questions that remain to be addressed. For instance, how do the initial surface properties of lithium impact electrochemical stability over long‐term cycling? To what extent does the lithiophilicity of the substrate shape the morphology and intrinsic chemistry of the solid electrolyte interphase (SEI)? Can researchers develop scalable wet‐processing techniques that are entirely contamination‐free yet still capable of producing high‐purity, conformal lithium films? Addressing these questions is essential for not only understanding the complex interdependencies between processing and performance, but also for guiding the rational design of next‐generation thin lithium metal anodes. In conclusion, this review goes beyond summarizing current fabrication methodologies by critically evaluating their limitations and the strategies used to overcome them. It provides a forward‐looking perspective on how future research efforts should be directed to overcome the persistent materials and processing challenges that hinder commercial LMB deployment.

## Conflict of Interest

The authors declare no conflict of interest.
